# Scale Drop Disease Virus Associated Yellowfin Seabream (*Acanthopagrus latus*) Ascites Diseases, Zhuhai, Guangdong, Southern China: The First Description

**DOI:** 10.3390/v13081617

**Published:** 2021-08-16

**Authors:** Yuting Fu, Yong Li, Weixuan Fu, Huibing Su, Long Zhang, Congling Huang, Shaoping Weng, Fangzhao Yu, Jianguo He, Chuanfu Dong

**Affiliations:** 1 School of Marine Sciences, Sun Yat-Sen University, Guangzhou 510006, China; fuyt6@mail2.sysu.edu.cn (Y.F.); zhanglong6@mail2.sysu.edu.cn (L.Z.); 2 Southern Marine Science and Engineering Guangdong Laboratory (Zhuhai), Zhuhai 519000, China; fuwx5@mail2.sysu.edu.cn (W.F.); lsswsp@mail.sysu.edu.cn (S.W.); 3 State Key Laboratory of Biocontrol, School of Life Sciences, Sun Yat-Sen University, Guangzhou 510275, China; 4 Zhuhai Modern Agriculture Development Center, Zhuhai 519000, China; ylshlee@gmail.com (Y.L.); huibing1111@163.com (H.S.); hcl008@126.com (C.H.); yf200033@163.com (F.Y.)

**Keywords:** scale drop disease virus, yellowfin seabream ascites diseases, genome, proteome, pathogenicity

## Abstract

Scale drop disease virus (SDDV), an emerging piscine iridovirus prevalent in farmed Asian seabass *Lates calcarifer* in Southeast Asia, was firstly scientifically descripted in Singapore in 2015. Here, an SDDV isolate ZH-06/20 was isolated by inoculating filtered ascites from diseased juvenile yellowfin seabream into MFF-1 cell. Advanced cytopathic effects were observed 6 days post-inoculation. A transmission electron microscopy examination confirmed that numerous virion particles, about 140 nm in diameter, were observed in infected MFF-1 cell. ZH-06/20 was further purified and both whole genome and virion proteome were determined. The results showed that ZH-06/20 was composed of 131,122 bp with 135 putative viral proteins and 113 of them were further detected by virion proteome. Western blot analysis showed that no (or weak) cross-reaction was observed among several major viral proteins between ZH-06/20 and ISKNV-like megalocytivirus. An artificial challenge showed that ZH-06/20 could cause 100% death to juvenile yellowfin seabream. A typical sign was characterized by severe ascites, but not scale drop, which was considerably different from SDD syndrome in Asian seabass. Collectively, SDDV was confirmed, for the first time, as the causative agent of ascites diseases in farmed yellowfin seabream. Our study offers useful information to better understanding SDDV-associated diseases in farmed fish.

## 1. Introduction

The family *Iridoviridae* is comprised of six genera, and is classified into two subfamilies: *Alphairidovirinae* and *Betairidovirinae* [1]. Members of *Alphairidovirinae* (*Ranavirus*, *Lymphocystivirus*, and *Megalocytivirus*) infect a variety of cold-blooded vertebrates. Among them, ranavirus infects bony fish, amphibians, and reptiles, whereas lymphocystivirus and megalocytivirus only infect bony fish. Members of *Betairidovirinae* (*Iridovirus, Chloriridovirus, and Decapodiridovirus*) infect invertebrates, including insects, crustaceans, and possibly mollusks [1]. Members of the family *Iridoviridae*, including large, icosahedral viruses, containing circular, double-stranded DNA genomes, with sizes ranging from 102 to 212 kb and consisting of 97 to 211 open reading frames (ORFs), can cause severe diseases, resulting in significant economic and environmental effects [2]. 

In recent years, megalocytiviruses have attracted great interest as they cause lethal systemic infections in wild and cultured freshwater, brackish, and marine bony fish worldwide [3,4]. Based on the nucleotide sequences of the viral major capsid protein (*mcp*) and adenosine triphosphatase (*ATPase*) genes, traditional megalocytivirus could be divided into three genotypes, and each genotype could be further subdivided into two separate subclades [5]: red sea bream iridovirus (RSIV), which has been reported in Japan, Korea, mainland China, Chinese Taiwan, and Southeast Asia, has caused serious economic loss in red seabream *Pagrus major* and many other marine and freshwater fish species [4,6]; infectious spleen and kidney necrosis virus (ISKNV), which has caused high mortality in mandarin fish *Siniperca chuatsi* in mainland China, has been reported as a major viral causative agent in tilapia *Oreochromis niloticus*, zebrafish *Danio rerio*, bluegill sunfish *Lepomis macrochirus*, and a variety of ornamental fishes [3,7,8,9]; and turbot reddish body iridovirus (TRBIV) mainly affects flatfish [5]. Complete genomic sequences of several ISKNV, RSIV, and TRBIV isolates have been determined and annotated, and the virion-associated proteins of both ISKNV-type and RSIV-type megalocytiviruses were identified by comprehensive proteomic approaches [5,10,11,12]. In these previous ISKNV-like megalocytiviral isolates, the GC content and genome sizes of these viruses ranged from 53% to 55% and 110,104 to 112,636 bp, respectively [5,11]. Genetic comparisons of the genome nucleotide sequences among different genotype isolates ranged from 93% to 98%. 

Scale drop syndrome (SDS) is a phenotypic symptom of diseased Asian seabass *Lates calcarifer.* The pathogen of SDS was firstly evidenced as a virus in 2012 and scientifically defined as a novel member of megalocytivirus in 2015, in Singapore, and the cumulative mortality was estimated at 40–50% [13,14]. Lesions mainly included scale loss, darkened bodies, tail/fin erosion, pallor of gills, and multifocal necrosis in the liver, spleen, and kidney. Severely affected fish were characterized by stopped schooling and sometimes spiral swimming. Histopathological findings observed vasculitis in all major organs and associated tissue degeneration, hemorrhage, and necrosis of varying severity, including the skin, heart, and spleen [14]. In the early 1990s, SDS was reported in Asian seabass in Penang, Malaysia. However, the cause of SDS remained unknown until 2015, when de Groof et al. identified and characterized a novel virus named scale drop disease virus (SDDV), by sequencing serum samples of scale drop syndrome-affected Asian seabass from Singapore. The virus was classified into the *Megalocytivirus* genus of the *Iridoviridae* family [13]. At present, the outbreaks of SDDV diseases have been widely prevalent in several Southeast Asian countries, including Singapore, Malaysia, Indonesia, and Thailand [13,15,16,17]. Moreover, the partial genome sequence of Singaporean SDDV isolate (accession no. NC027778) and the whole genome sequence (accession no. MN562489) of Thailand isolate were determined as 124,244 bp and 131,129 bp, comprised of 129 ORFs and 135 ORFs, respectively [13,15]. A blastn search showed that the Singaporean SDDV identity was low with previous ISKNV/RSIV/TRBIV-like megalocytiviruses, at most 60% depending on the core viral gene. In addition, an SDDV-close European chub iridovirus (ECIV), was isolated and characterized from European Chub *Squalius cephalus* in 2019, England. The complete genome sequence of ECIV was 128,216 bp, encoding a total of 108 ORFs, and the *ATPase* and *mcp* nucleotide, identified to previous ISKNV/RSIV/TRBIV-like megalocytiviruses, ranged from 66.4 to 76.9% and 62.8 to 73.1%, respectively [18]. 

Yellowfin seabream *Acanthopagrus latus* is a commercially and ecologically important species and widely distributed throughout the Indo-West Pacific. In view of its high market demand, stable price, and high breeding profit, yellowfin seabream has become one of the most important economic fish in South China around the coastal area. Yellowfin seabream is suitable for aquaculture in brackish and fresh water areas and usually inhabits warm shallow and coastal waters [19]. In recent years, diseases with severe ascites were observed frequently in cultured yellowfin seabream, in Zhuhai city of Guangdong province, where the Golden Bay Yellowfin Seabream Guangdong Provincial Modern Agricultural Industrial Park is established. The causative agent of yellowfin seabream ascites diseases (YFSBAD) has remained unclear for several years. 

Prior to this study, SDDV infections were only reported in farmed juvenile and adult Asian seabass in several SE countries [16]. An SDDV-close ECIV infection was only found in the European chub in England [18]. In the present study, an SDDV isolate ZH-06/20 was isolated from yellowfin seabream ascites farmed in Zhuhai, South China. ZH-06/20 was characterized by cell culture, transmission electron microscope, whole genome, virion proteome, and pathogenicity. Our study evidenced that yellowfin seabream was the third natural host fish species for SDDV iridovirus.

## 2. Materials and Methods

### 2.1. Fish Sampling and Virus Isolation

Ascites diseases occurred in a yellowfin seabream farm located in Jinwan district, Zhuhai city of Guangdong province, China. Five sample fish, ranging from 11 to 15 g and body lengths of about 16 cm, were collected for pathogen isolation and identification. The clinical signs of diseased fish included swollen abdomens with severe ascites, splenomegaly, and petechial to ecchymotic hemorrhage in the liver. The fish were carefully dissected and the liver, spleen, kidney, and ascites were collected with sterile scissors and tweezers. The ascites was centrifuged at 7500× *g* for 10 min at 4 °C and then filtrated through a 0.22 μm membrane (Millipore, Burlington, MA, USA). The filtrated ascites supernatant was used to inoculate with mandarin fish fry cells for virus isolation. 

Mandarin fish fry (MFF-1) cell line was established and characterized in our laboratory, grown in ambient air with 5% CO_2_ at 26 °C within Dulbecco Modified Eagle Medium (DMEM) supplemented with 10% fetal bovine serum (FBS) (Gibco Invitrogen) to obtain monolayer cells [20]. For virus isolation, 200 μL of filtered ascites supernatant was added to a 10-cm diameter of tissue culture dish with 10 mL DMEM. The inoculated MFF-1 cells were observed daily under an inverted microscope. When MFF-1 cells exhibited apparent cytopathic effect (CPE) up to 80%, the infected cells were collected, stored at −80 °C, frozen and thawed for three cycles. The yielded virus was labeled as virus passage 1. The cell suspension was used for another round infection and the yielded virus was labeled as virus passage 2, followed by virus passage 3, virus passage 4, and so on. Virus passage 8 was used for further virus identification and an experimental challenge. The isolated virus was designated as ZH-06/20. 

### 2.2. Virus Purification and Genomic DNA Extraction

When MFF-1 cells were confluent, ZH-06/20 with a multiplicity of infection (MOI) of approximately 2.0 was added to 75 cm^2^ tissue flasks. Infected cells were harvested at about 70% complete CPE, usually at 3–4 days post-infection (dpi), stored at −80 °C, followed by three cycles of freezing/thawing. To purify ZH-06/20, the cell suspension was treated by differential centrifugation, ultracentrifugation, and double sucrose density gradient centrifugation, as previously described by Dong et al. [10]. Briefly, ZH-06/20-infected MFF-1 cells were collected and frozen and thawed for three cycles. Suspensions were centrifuged at 8000× *g* for 40 min at 4 °C. The supernatant was then centrifuged at 150,000× *g* for 1 h at 4 °C. The pellet was resuspended in sterile phosphate buffered saline (PBS, pH 7.4) and the virus suspensions were overlaid on 35% sucrose and centrifuged at 150,000× *g* for 1 h at 4 °C. The pellet was resuspended in PBS and then reloaded on 30%–60% linear sucrose gradients (Bio-Rad) for ultracentrifugation at 150,000× *g* for 1 h at 4 °C. Finally, the visible bands were extracted carefully. The purified virus was resuspended in sterile PBS, examined by transmission electron microscopy (TEM), and used for preparation of the genomic DNA, construction of libraries, and virion proteomic analysis.

Genomic DNA from purified ZH-06/20 was prepared by using NucleoSpin Tissue XS (MACHEREY-NAGEL, Neumann, Neander, Germany), according to the manufacture’s protocol. The harvested DNA was detected by agarose gel electrophoresis and quantified by Qubit2.0 Fluorometer (Thermo Scientific, Waltham, MA, USA). 

### 2.3. Transmission Electron Microscope (TEM)

In order to observe the basic morphological structure of viral particles, ZH-06/20-infected MFF-1 cells at 3 dpi were collected for the TEM assay. TEM analysis was performed as described in a previous study [10]. Ultrathin sections were stained with uranyl acetate-lead citrate and examined under a JEOL JEM-1400 electron microscope (Japan). 

### 2.4. Library Construction, Sequencing, and Genome Assembly 

A total of 1 μg of viral DNA was prepared to the construct library. The sequencing library was generated using NEBNext® Ultra™ DNA Library Prep Kit for Illumina (NEB, Ipswich, MA, USA) following the manufacturer’s recommendations. Briefly, the DNA sample was fragmented by sonication to a size of 350 bp, then DNA fragments were end-polished, A-tailed, and ligated with the full-length adaptor for Illumina sequencing with further PCR amplification. Finally, PCR products were purified (AMPure XP system) and libraries were analyzed for size distribution by the Agilent 2100 Bioanalyzer and quantified using real-time PCR. 

The whole genome of ZH-06/20 was sequenced using Illumina NovaSeq PE150 at the Beijing Novogene Bioinformatics Technology Co., Ltd. (Beijing, China).The raw data obtained by sequencing (raw data) was filtered to obtain valid data (clean data) in order to ensure the accuracy and reliability of the subsequent information analysis results. At the same time, host-related DNA was filtered by mapping clean reads against the mandarin fish genome (accession no. GCA_011952085.1) using Bowtie2 to retrieve the unmapped reads.

The clean data were used for genome assembly with SOAPdenovo (Version 2.04), SPAdes and AbySS software. The assembly results were integrated with CISA software, and optimized with GapCloser software (Version 1.12) to obtain the final assembly results.

### 2.5. Genome Functions, Structure Prediction and Phylogenetic Tree Construction

The gene functions and structures were predicted based on BlastP searches against the National Center for Biotechnology Information (NCBI) and the Simple Modular Architecture Research Tool (SMART) website, http://smart.embl-heidelberg.de/ (accessed on 17 March 2021). The presumptive amino acid sequences were submitted to the NCBI network service to search for conserved domains, motifs, or signatures from the NCBI CD-Search database. The mcp gene sequences of 34 iridoviruses including ZH-06/20 were aligned using ClustalX, and a phylogenetic tree was constructed by the neighbor-joining method using MEGA (Version 5.0) software, with 1000 bootstrap replicates. 

### 2.6. Antibody Preparation 

Primer sets for the ZH-06/20 *mcp* gene were designed according to the ZH-06/20 genomic sequence. The primers were MCP-F (BamHI) 5′CGGGATCCATGTCATCTATTGC AGGAGCTAATG3′ and MCP R (HindIII) 5′CCCAAGCTTCAAGATCGGAAATCCAAATGA 3′. Standard PCR and molecular biology protocols were used to amplify the *mcp* gene using purified ZH-06/20 genomic DNA as a template. The purified PCR product was cloned into plasmid pMal-c2X to generate the pMal-c2X-MCP plasmid. The recombinant plasmid was confirmed by sequencing and then expressed in *Escherichia coli* BL21. Overnight cultures of *E. coli* BL21 harboring recombinant plasmid were diluted to 1:100 (*vol*/*vol*) in fresh Luria–Bertani broth supplemented with ampicillin (100 μg/mL) and incubated at 37 °C until the optical density (OD600) reached 0.6–0.8. A final concentration of 1 mM/liter isopropyl-β-d-thiogalactopyranoside (IPTG) was added in bacteria and incubated for 6 h at 37 °C to induce expression of the MBP-MCP fusion protein. The expressed protein was then detected by SDS-PAGE. Bacterial cells were harvested by centrifugation at 6000 rpm for 10 min and the bacterial pellet was resuspended in precooled-PBS for high pressure crushing. After centrifugation (9000 rpm for 12 min at 4 °C), the supernatant and the sediment resuspended in PBS were subjected to SDS-PAGE, the rest of the supernatant and sediment were stored at −80 °C and protein concentrations were determined by the TaKaRa BCA Protein Assay Kit (TaKaRa, Kusatsu, Shlga, Japan), according to the protocol. The gel was stained in Coomassie Blue staining (0.1% Coomassie Brilliant Blue R-250, 25% isopropanol, 10% glacial acetic acid) and then destaining in decolorizing solution (10% acetic acid, 5% ethanol) until the protein bands were visible clearly. The gel was then washed in distilled water for 24 h and a spot of MBP-MCP fusion protein was manually excised from the gel, followed by grinding with sterile PBS. 

The grinded gel (containing 1 mg MBP-MCP fusion protein) was emulsified with equal volumes of Freund’s complete adjuvant (FCA) for the first immunization by subcutaneous injection (i.s.) and Freund’s incomplete adjuvant (FIA) for the following three booster injections. New Zealand rabbit was received four i.s. immunizations at 2-week intervals. Two weeks after the final injection, the rabbit was bled for serum collection and the serum were stored at −80 °C until use. Animal work was approved by Institutional Animal Care and Use Committee, Sun Yat-sen University. The approved number was SYSU-IACUC-2021-000324.

### 2.7. Western Blotting Assay

Western blot assay was used to test the effectiveness of the prepared anti-recombinant ZH-06/20 MCP and to further assess the possible cross-reaction between SDDV and ISKNV. Several poly-antibodies (pAbs) of rabbit anti-recombinant viral structural proteins of ISKNV-MCP, VP007, and VP101 were presented in our previous report [10]. pAb of a non-structural ISKNV-VP023 was referred to by Xu et al. [21]. Mouse monoclonal antibodies (mAbs) against ISKNV-2D8 and VP023 were prepared and stored by our team (unpublished data by Dong et al.). For western blot analysis, these antibodies were diluted with suitable dilution (1:1000–2000) to use as the first antibodies to recognize the viral protein. HRP-conjugated goat anti-rabbit or anti-mouse IgG was used as the secondary antibody, and the blot was visualized by addition of the Tanon High-sig ECL Western Blotting Substrate (Tanon, Shanghai, China). 

### 2.8. Virion Proteome by LC–MS/MS

The concentration of purified viral proteins was determined; the viral proteins were further analyzed by LC–MS/MS as previously described [10,12]. Briefly, SDT lysis buffer (4% SDS, 100 mM Tris-HCl, 1 mM DTT, pH 7.6) was added to 1 µg of purified virion protein resuspended in sterile PBS. The lysate was boiled for 15 min and the supernatant was stored at −80 °C after centrifuged at 14,000× *g* for 40 min. Dithiothreitol (DTT) was added to the lysate, to a final concentration of 10 mM for reduction of proteins, and then the lysate was incubated at 37 °C for 1.5 h. For alkylating proteins, iodoacetamide (IAA) was then added to a final concentration of 50 mM followed by incubation at room temperature in the dark for 40 min. Afterward, trypsin was added (a ratio of trypsin to protein at 1:50 (*w*/*w*)) for digestion at 37 °C overnight. Trifluoroacetic acid was added to a final concentration of 1% to stop trypsin digestion. The peptides of ZH-06/20 were desalted on C18 Cartridges (Empore™ SPE Cartridges C18, bed I.D. 7 mm, volume 3 mL, Sigma, Saint Louis, MO, USA), concentrated by vacuum centrifugation and reconstituted in 40 µL of 0.1% (*v*/*v*) formic acid. The peptides obtained after digestion were subjected to nano LC–MS/MS analysis in the Shanghai Applied Protein Technology Co., Ltd. (Shanghai, China). The acquired MS/MS spectra were searched using MASCOT engine (Matrix Science, London, UK; Version 2.4).

### 2.9. Artificial Challenge

Virus passage 8 was used for an artificial infection experiment. Before infection, five juvenile yellowfin seabream were randomly sampled for SDDV and ISKNV detection by conventional PCR; they were virus-free. Forty juvenile yellowfin seabream (about 3 g) were used for artificial infection. Before infection, all fish were kept for 7 days to adapt to the environment and then divided into two groups, with 20 fish per group. One group was intraperitoneally injected with 0.1 mL of the virus (10^3.5^ TCID_50_/fish), and another group was injected with 0.1 mL of sterile PBS as an “un-infection” control. The fish were monitored daily to calculate the morbidity and mortality until there were no fish deaths for 5 days. Moribund fish were collected, and liver, spleen, and kidney tissues were prepared for histopathology study. 

### 2.10. Histopathology and Immunofluorescence Assay (IFA)

Moribund fish infected with ZH-06/20 were sampled; the livers, spleens, kidneys, brains, gills, and muscles were dissected and fixed with alcohol–formalin–acetic acid (AFA) for hematoxylin–eosin (H&E) staining, or fixed with 4% paraformaldehyde for immunofluorescence assay (IFA) slices. The tissue sections were made according to protocols described previously and used for histopathology with H&E staining and antibody-based IFA analysis, respectively [9]. 

For IFA, ZH-06/20 MCP pAb and AlexaFluor488-conjugated (green fluorescence) goat anti-rabbit IgG (Abcam, Shanghai, China) were used as the primary and secondary antibodies, respectively. The nucleus was stained by Hoechst 33342 (Invitrogen, Waltham, MA, USA). Sections were visualized under a fluorescence microscope microscopy (Nikon, Tokyo, Japan).

## 3. Results

### 3.1. Mortality Events and Clinical Symptoms

For several years, ascites diseases have occurred (and are still prevalent) in farmed yellowfin seabream in Zhuhai, Guangdong province. The most obvious clinical symptom of the diseased fish is a swollen abdomen with severe ascites; the local famers call the disease yellowfin seabream ascites diseases (YFSBAD). The body sizes of infected yellowfin seabream range from small-sized juvenile to large-sized growing fish. 

**Figure 1:**
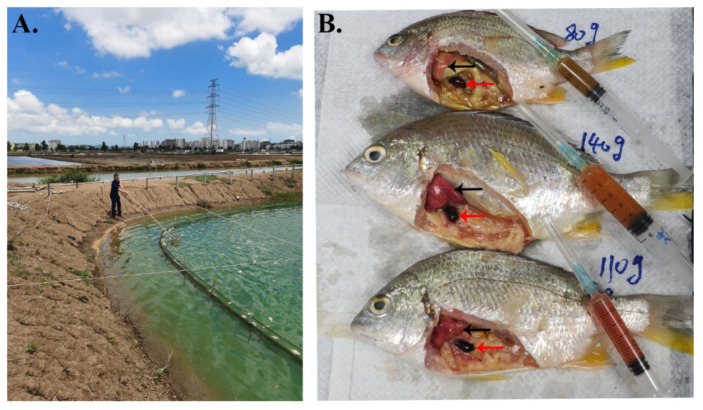
A recent mortality event of YFSBAD and the clinical symptoms of naturally infected large-sized yellowfin seabream (80~140 g per fish). (**A**) A recent outbreak of YFSBAD in a local farm in Zhuhai. (**B**) The diseased fish were characterized by swollen abdomens, ascites, splenomegaly, and petechial to ecchymotic hemorrhage in the liver. Over 2 mL of ascites were extracted from each fish. The red arrows indicate enlarged spleens. The black arrows indicate bloodless liver.

In particular, the outbreaks of YFSBAD usually occur in outgrowing yellowfin seabream. For example, while preparing this manuscript, a recent outbreak of YFSBAD was monitored in a local yellowfin seabream farmed in Jinwan district, Zhuhai. A total of 100,000 growing fish with body weights ranging from 80 to 140 g per fish were cultured in one pond (Figure 1A). During outbreak of YFSBAD, within one month, about 60,000 fish died of severe ascites. The diseased fish were dissected, and abundant ascites were drawn (Figure 1B). Additionally, swollen spleens and bloodless livers were observed (Figure 1B). In this case, an SDDV was isolated and confirmed by cell culture and conventional PCR detection using ascites as sample resources (data not shown). No obvious scale drop symptoms (SDS) were observed on the body surfaces of these diseased fish, which were considerably different from the typical symptoms of Asian seabass infected by SDDV in other SE countries [13,17].

### 3.2. Virus Isolation 

**Figure 2:**
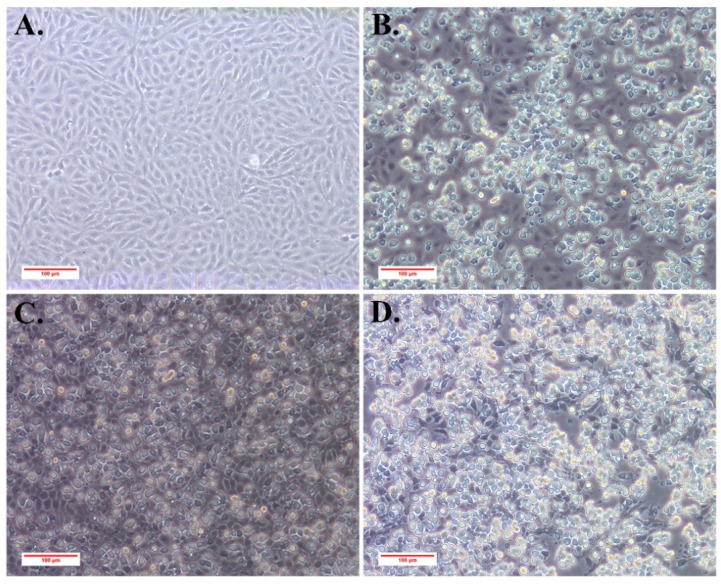
Replication of ZH-06/20 in MFF-1 cells. (**A**) Normal MFF-1 cells; (**B**) ascites-inoculated MFF-1 cells on day 6 post-inoculation; (C/D) ZH-06/20 at passage 2 (**C**) and passage 7 (**D**) in MFF-1 cells at 2 days post-infection.

The filtered ascites supernatant was used to inoculate directly into MFF-1 cells. As a result, advanced CPEs were observed 6 days post-inoculation (Figure 2B). The CPE was characterized by an increasing amount of rounding cells, similar to that of ISKNV-like megalocytivirus infection, but not similar to that of MRV-like ranavirus infection [22]. The infected MFF-1 cells were harvested at −80 °C and labeled as virus passage 1. After three frozen/thaw cycles, virus passage 1 was used for another round infection. Since virus passage 3, ZH-06/20 could induce complete CPE in MFF-1 cells and the viral titers were determined as 10^6.6~7.2^ TCID_50_/0.1 mL. Figure 2C,D indicate the virus passage 2 and virus passage 7 virus in MFF-1 cells at 2 days post-infection, respectively (Figure 2C,D). ZH-06/20, at passage 7, was more infectious to MFF-1 cells than that of virus passage 2. ZH-06/20 has been passaged in MFF-1 cells over 30 times. More SDDV-like viruses, except for ZH-06/20, have been isolated in MFF-1 cells. These data show that MFF-1 is a highly sensitive cell line suitable for effective isolation of SDDV.

### 3.3. Transmission Electron Microscope (TEM)

**Figure 3:**
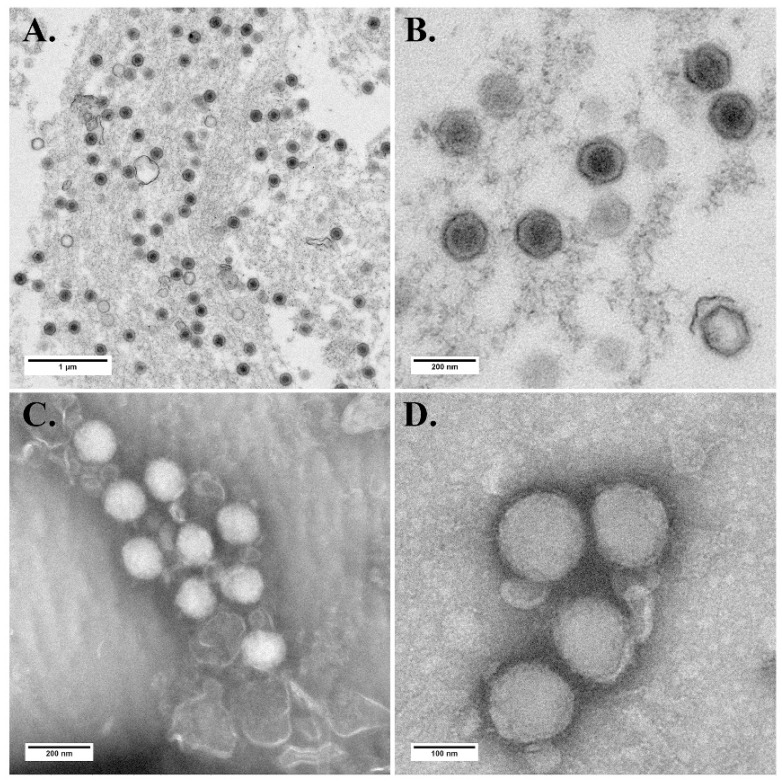
Transmission electron photomicrograph of ZH-06/20-infected MFF-1 cells and purified viral particles from infected MFF-1 cells. (**A**,**B**) Numerous hexagonal viral particles with a diameter of about 140 nm were observed in infected MFF-1 cells with low and large magnifications. (**C**,**D**) Spherical viral particles purified from infected MFF-1 cells with low and large magnifications.

ZH-06/20-infected MFF-1 cells and purified ZH-06/20 virions were examined under TEM. Results show that numerous hexagonal viral particles, with a diameter of 140 nm, were observed in the cytoplasm of the infected MFF-1 cell (Figure 3A,B). Intact spherical virions were also observed by TEM examination (Figure 3C,D). The results suggest that ZH-06/20 could be proliferated well in MFF-1 cells, as both ISKNV-like and RSIV-like megalocytiviruses have done [20,23]. 

### 3.4. Whole Genomic Sequence of ZH-06/20 

**Figure 4:**
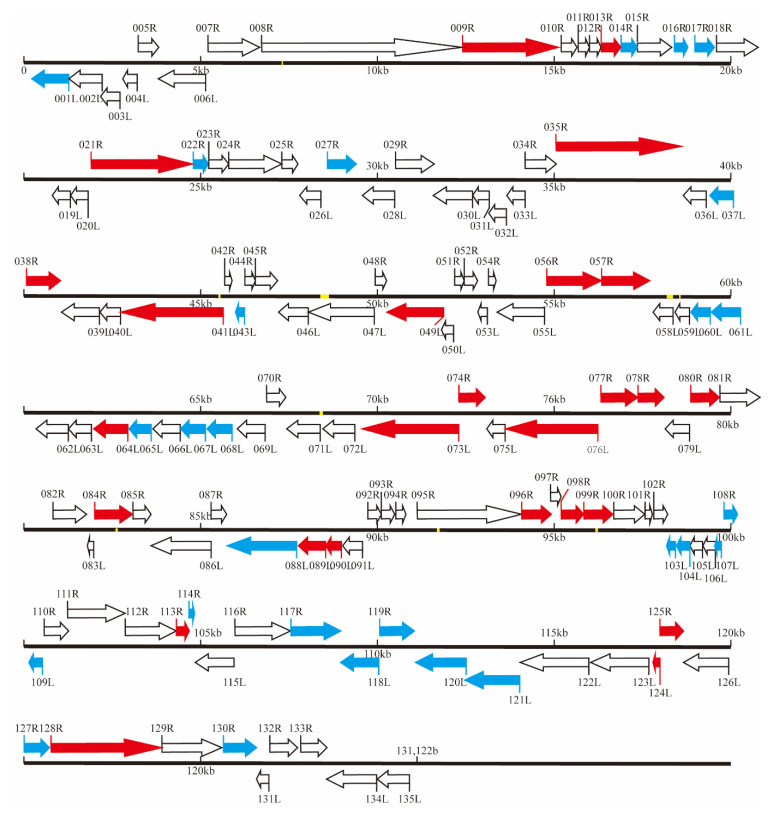
Liner schematic organization of ZH-06/20 genome. The 26 common core genes in iridovirus, 27 ZH-06/20 specific genes and ZH-06/20 tandem repeat areas are indicated in red, blue, and yellow, respectively. Generally, ZH-06/20 is composed of 131,122 nucleotides with 135 ORFs and 27 unique genes are never found in other iridoviruses, except for SDDV.

**Figure 5:**
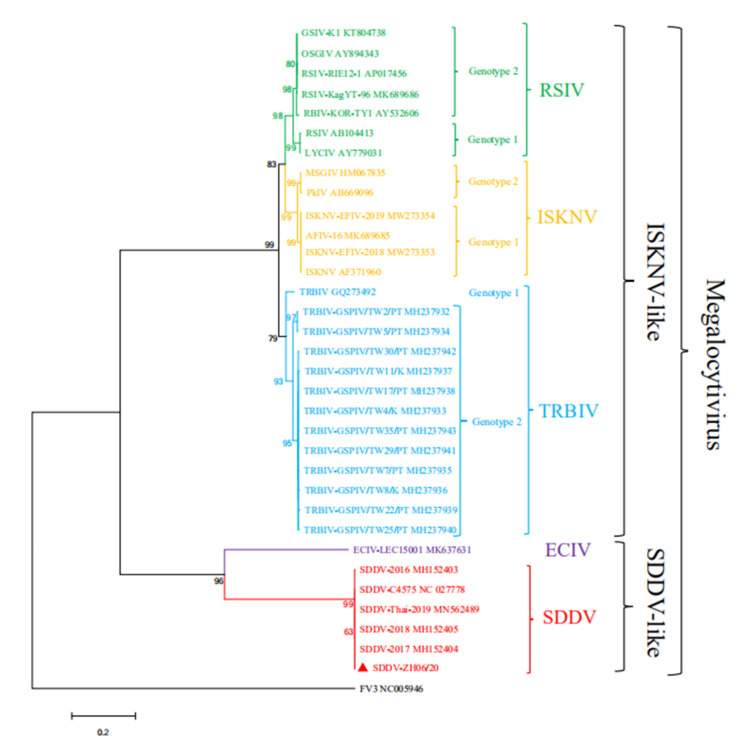
Phylogenetic relationship of ZH-06/20 and 32 other members in the genus *Megalocytivirus* based on the *mcp* gene. A red solid triangle indicated the ZH-06/20. The FV3 in genus *Ranavirus* was used as an outgroup. Generally, megalocytivirus could be divided into two subgroups. The ISKNV-like subgroup includes RSIV, ISKNV, and TRBIV and the SDDV-like subgroup includes SDDV and ECIV. Genomic sizes of viral members in ISKNV group are about 111,000 bp. By contrast, genomic sizes of members in the SDDV-like group are 128,000~132,000 bp. FV3 in genus *Ranavirus* was used as an outgroup.

The whole genome of ZH-06/20 was determined using an Illumina sequencer. The result show that whole genome of ZH-06/20 consisted of 131,122 bp with 135 putative ORFs (Figure 4), with predicted molecular masses ranging from 6.42 to 211.7 kDa (Table S1) and a G + C content of 36.56%, which was considerably different from that of ISKNV (54.78%), but similar to SDDV (37%) and ECIV (38.83%). Comparative genomic analysis revealed that ZH-06/20 also contained 26 iridovirus core genes [24] (Figure 4). Comparison between this genome and the Singaporean SDDV (accession no. NC_027778.1) and Thailand SDDV (accession no. MN562489.1) revealed that the nucleotides identified within the aligned regions were 99.94% and 99.91%, respectively. The *mcp* nucleotide sequences of the three isolates were completely identical. However, the nucleotide sequence of ZH-06/20 *mcp* has no significant or low similarity to that of ISKNV and ECIV. At the amino acid sequence level, the similarity of ZH-06/20 MCP to that of ECIV and ISKNV was 81.06 and 68.57%, respectively. Phylogenetic analysis based on *mcp* gene sequences showed that the ZH-06/20 was clustered into the SDDV-like clade in the genus *Megalocytivirus*, but it separated from the clades of ISKNV, RSIV, and TRBIV (Figure 5). Conserved domains, motifs, or signatures identified from the NCBI CD-Search database, and the gene structure and function (predicted through the website http://smart.embl-heidelberg.de/ (accessed on 17 March 2021)) are showed in Table S1. The whole genome of ZH-06/20 had 12 repetitive regions throughout the genome. In total, these occupied 0.64% of the ZH-06/20, varying in sizes from 12 to 117 bp.

### 3.5. The Profiles of Virion Proteome

**Figure 6:**
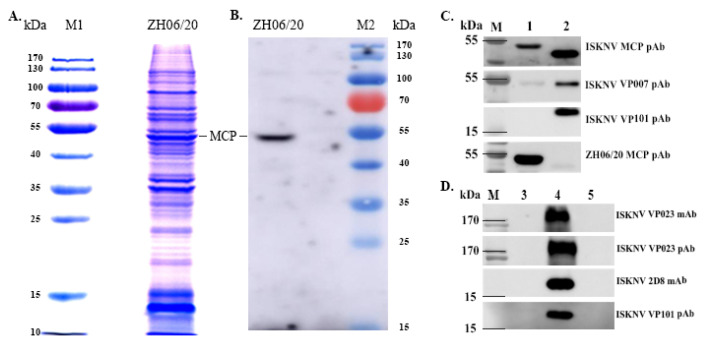
(**A**,**B**) Viral protein of the purified ZH-06/20. (**A**) Protein profile of the crude purification of ZH-06/20 by SDS-PAGE. (**B**) Western blot analysis of ZH-06/20 proteins recognized by anti-recombinant MCP pAb of ZH-06/20. (**C**,**D**) Cross-reaction between ZH-06/20 and ISKNV using different anti-ISKNV and anti-ZH-06/20 antibodies by western blot analysis. (**C**) The purified ZH-06/20 and ISKNV virions were recognized by anti-ISKNV-MCP, -VP007, -VP101, and anti-ZH-06/20 MCP pAbs. (**D**) ZH-06/20 and ISKNV-infected MFF-1 cells were recognized by anti-ISKNV VP101 and VP023 pAbs and anti-ISKNV 2D8 and VP023 mAbs. M: marker; Lane 1–2: Purified ZH-06/20 and ISKNV; Lane 3–5: ZH-06/20, ISKNV, and mock infected-MFF-1 cells, respectively. pAb, poly-antibody; mAb, monoclonal antibody.

SDS-PAGE analysis indicated that there were over 30 obvious protein bands in the gel stained by Coomassie Brilliant Blue R-250 (CBB-R-250) (Figure 6A), and the most abundant viral protein, the major capsid protein, could be recognized by anti-ZH-06/20 rMCP antibody (Figure 6B). The purified virions were further analyzed by LC–MS/MS. A total of 113 proteins were identified, among which, 100 proteins had at least two unique peptides, indicating a significant confident identification (Table S2). To assess the possible cross-reaction between ZH-06/20 and the traditional ISKNV-like megalocytivirus. pAbs against three well-characterized ISKNV structural proteins, namely the MCP (a highly conserved and the highest of the abundant amount of viral proteins in iridovirus), VP007 (a highly conserved and highly abundant viral myristylated envelope protein), and VP101 (a highly abundant small viral structural protein) were used to identify both ZH-06/20 and ISKNV-NH060831 [10]. Furthermore, both pAb and mAb, against a well-studied non-structural protein of ISKNV-VP023 [21], and mAb against ISKNV 2D8, were also used as the first antibodies to differently recognize ZH-06/20 and ISKNV. The results showed that both pAbs against ZH-06/20 and ISKNV-rMCP could strongly recognize their respective MCPs, but slightly reacted with the opposite MCPs (Figure 6C). pAb of ISKNV-VP007 also slightly reacted with a viral protein in ZH-06/20. No cross-reactions were observed between ZH-06/20 and ISKNV-NH060831, no matter what pAbs against ISKNV-VP101 and VP023 and mAbs against ISKNV-2D8 and VP023 were used as the first antibodies (Figure 6D). 

### 3.6. Pathogenicity to Juvenile Yellowfin Seabream

**Figure 7:**
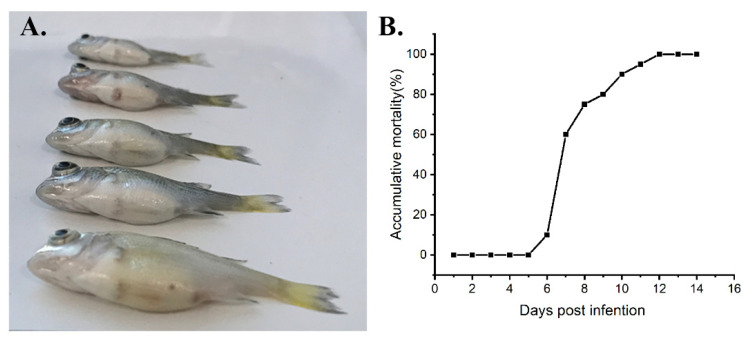
Infectivity of ZH-06/20 to juvenile yellowfin seabream under artificial conditions. (**A**) The infected fish under artificial conditions showed ocular proptosis and swollen abdomens. (**B**) The mortality of artificial-infected yellowfin seabream was observed on the 6th day post-challenge.

To confirm whether ZH-06/20 is the causative agent of YFSBAD, artificial infection was performed under laboratory conditions. The artificial infection was applied via intraperitoneal injection (10^3.5^ TCID_50_/fish). The morbidity and mortality were observed on the 6 dpi and the cumulative mortality reached to 100% on the 12th day post-infection (Figure 7B). No mortality was observed in the uninfected control group. The typical clinical symptom of moribund fish was also characterized by swollen abdomens with ascites (Figure 7A), which was similar to that of the natural outbreak of YFSBAD (Figure 1B), suggesting that ZH-06/20 would be the causative agent of YFSBAD. 

### 3.7. Histopathological and IFA Observation

**Figure 8:**
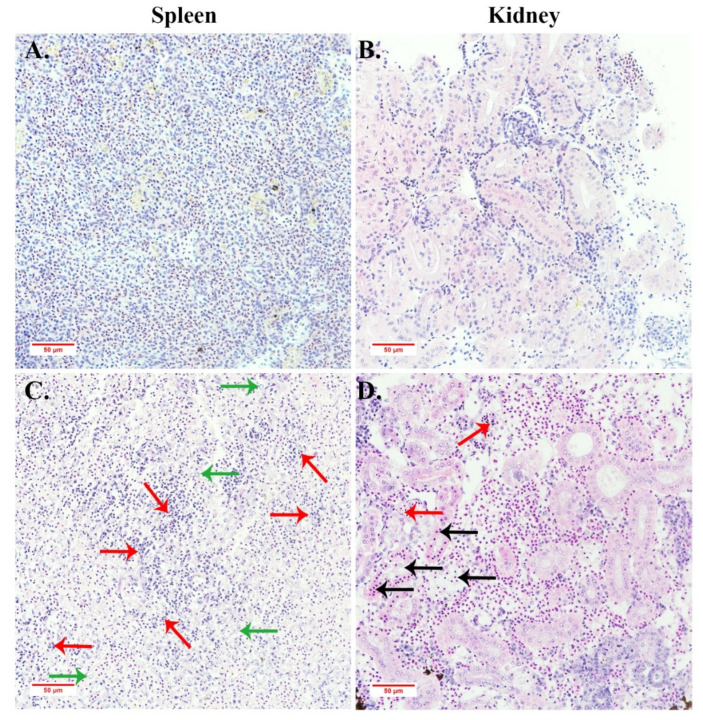
Histopathological observation of the spleen and kidney of ZH-06/20-infected yellowfin seabream. (**A**,**B**) were the normal spleen and kidney, respectively. (**C**) Infected spleen tissue showed the most severe lesions with abundant vacuolated cells (green arrows) and diffuse karyolysis (red arrows). (**D**) Infected kidney tissue exhibited some karyolysis (red arrows) and distinct pyknosis (black arrows).

Histopathological examination showed that the infected spleen and kidney tissues had remarkable histopathological changes (Figure 8). Numerous vacuolated cells with diffuse karyolysis were observed extensively in infected spleen tissues, which suggested multifocal areas of splenic necrosis. Similarly, there were a few karyolysis cells, but significant pyknosis observed in the infected kidneys. Tissue cells were loosely arranged in the spleen and kidney tissues, in contrast, the pathological changes of the liver were not so evident (data not shown).

**Figure 9:**
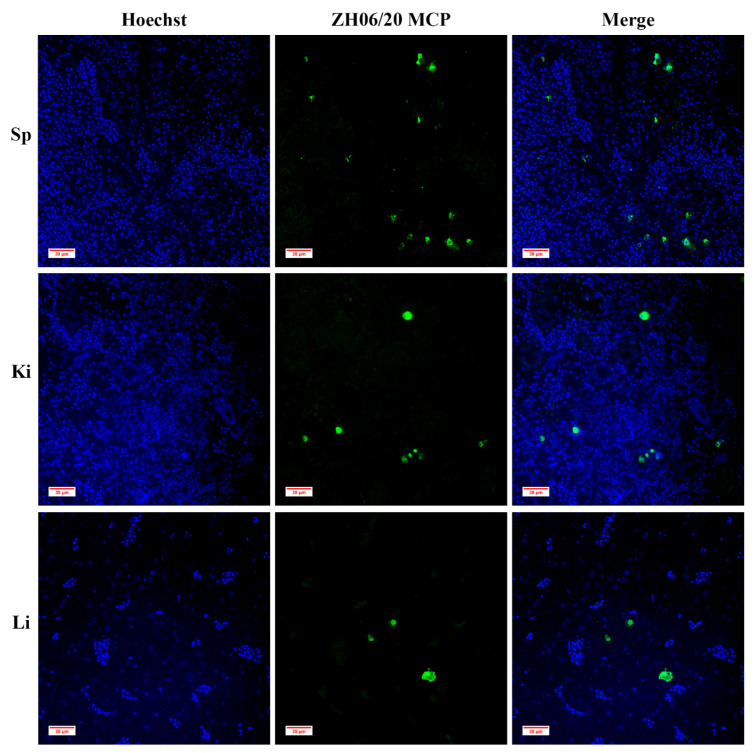
Immunofluorescence observation of infected tissues of spleen (Sp), kidney (Ki) and liver (Li). The infected cells are labeled by green fluorescence, which are associated with anti-ZH-06/20 MCP. Strongest fluorescence signals are observed in the spleen, then in the kidney and liver.

Strong fluorescence signals were observed in the spleen, kidney, and liver using immunofluorescence assay (Figure 9); this is highly consistent with the observed histopathological examination findings.

## 4. Discussion

Since red seabream iridovirus (RSIV) was firstly documented in cultured red seabream in Shikoku Island, Japan, in 1990 [25], and the genus *Megalocytivirus* was defined in 2005 [26]; in the past 30 years, megalocytivirus has become a worldwide threat to extensive farmed freshwater and marine bony fish distribution in Asia, Europe, America, Africa, and Australia [3,5,27,28]. The traditional megalocytiviruses were composed of three classic clades, namely RSIV, ISKNV, and TRBIV, and a further six subclades of RSIV-I and RSIV-II, ISKNV-I and ISKNV-II, and TRBIV-I and TRBIV-II. Among them, ISKNV was defined as a type species due to a series of well-characterized studies. In general, the genome content, pathogenicity, viral antigen, histopathology, as well as the general diagnosis methods of RSIV, ISKNV, and TRBIV have high similarity [12,24]. By contrast, the emerging SDDV is a distinct member of the genus *Megalocytivirus*. 

A previous study confirmed the causative agent of SDS as a distinct member of megalocytivirus in diseased *L. calcarifer* in Singapore, in 2015, through comprehensive cell culture-based virus isolation, TEM observation-based virus identification, and complete genome determination-based virus taxonomic [13]. The virus was designated as scale drop syndrome virus (SDDV) and defined as a novel member in the genus *Megalocytivirus* [13]. Nowadays, SDDV was widely prevalent in cultured *L. calcarifer* in extensive SE countries, including Singapore, Malaysia, Indonesia, and Thailand [13,15,16,17]. In addition, an SDDV-close ECIV was recently reported in European chub in England [18]. Comparative genome analysis showed that SDDV isolates in SE countries have almost the same genome content and obvious differences from that of ECIV, indicating that SDDV and ECIV might have a different evolution origin. SDDV or SDDV-like viruses have never been documented in any other fish species (except for SDDV in *L. calcarifer* in SE countries and ECIV in European chub). Compared with the well-studied ISKNV-like traditional megalocytivirus—the SDDV-like virus contained larger genome content, caused different histopathology, and contained too many mysterious veils to be revealed.

In this study, SDDV ZH-06/20 was isolated from yellowfin seabream ascites. To the best of our knowledge, yellowfin seabream was the third natural host fish species for SDDV iridovirus. The clinical symptoms of ZH-06/20-infected yellowfin seabream are characterized by swollen abdomens with severe ascites, splenomegaly, petechial to ecchymotic hemorrhage in the liver, and ocular proptosis (Figure 1B and Figure 7A), which are considerably different from those of SDDV-infected *L. calcarifer*, characterized with scale loss, darkened bodies, tail and fin erosion, and gill pallor [13,14,16]. The causes of such various clinical and histopathological differences by (nearly) the same virus require further study. 

At the genome level, the whole nucleotide identities among ZH-06/20 and the Singaporean isolate (accession no. NC_027778.1) and the Thailand isolate (accession no. MN562489.1) are 99.94% and 99.91%, respectively. Minor differences also exist among these isolates. For example, compared with the Thailand SDDV genome, 80 nucleotides were missed between ZH-06/20 ORF 72 and ORF 73, whether these deletions affect the pathogenicity or determine cross-host transmission will be studied further. Based on the most conserved *mcp* gene, phylogenetic analysis showed that megalocytivirus could be divided into two clusters, namely an ISKNV-like and SDDV-like clade, respectively (Figure 5). ZH-06/20 was clustered into an SDDV-like clade and had very high similarity in the whole genome content and the ORF component with that of SE *L. calcarifer* SDDV isolates, rather than that of the ECIV isolate in England. We have strong reasons to speculate that SDDV is likely to spread transboundary from SE countries, but not from England to mainland China by some unknown routes. In particular, it is worth mentioning that *L. calcarifer* is the only known host fish species for natural SDDV infection in SE countries [16]. Although *L. calcarifer* is the major farmed fish species in the same area in Zhuhai, Guangdong, no “scale drop disease” case had been documented in the past several years. On the contrary, we recently exhibited strong evidence that the ISKNV-II; genotype megalocytivirus was the causative agent for mass mortality of juvenile farmed *L. calcarifer* in Zhuhai [9]. In Zhuhai, Guangdong, the local seabass *Lateolabrax maculatus*, Asian seabass *Lates calcarifer*, and yellowfin seabream *Acanthopagrus latus* are the three major farmed fish species with commercial fish products, greater than 120,000, 80,000, and 15,000 tons each year, respectively. In some large-scale farm companies, the three fish are even cultured in different adjacent ponds in the same farm. Confusingly, the yellowfin seabream is the only natural host fish for this SDDV pathogen. Future studies will be performed to investigate and assess whether SDDV could disseminate from *Acanthopagrus latus* to *Lateolabrax maculatus* and *Lates calcarifer* in Zhuhai. Thus, if *L. calcarifer* is also a sensitive fish species, in regard to ZH-06/20 infection, would scale drop syndrome (but not ascites) be the featured clinical sign?

In a traditional ISKNV-like clade, effective inactivated whole cell vaccines of RSIV and ISKNV have been developed and licensed in Japan and China, respectively [29,30,31]. A previous study showed that inactivated RSIV vaccine conferred no cross-protection against SDDV in a *L. calcarifer* model [13]. In piscine iridovirus, a certain degree of antigenic cross-reactions could be observed between different genera, for example between ranavirus and RSIV-like megalocytivirus [32]. No cross-protection was observed in inactivated ISKNV vaccine immunized mandarin fish against MRV infection [31], although the inactivated ISKNV vaccine provided the same effective protection against both ISKNV- and RSIV-type megalocytiviruses [33]. In this study, virion proteins of ZH-06/20 were analyzed, and a total of 113 viral proteins were identified, among which, 100 viral proteins had confident identification via the LC–MS/MS approach (Table S2). The identified viral proteins of purified ZH-06/20 were remarkable—more than those of ISKNV and RSIV [10,12]. The combined identified viral proteins, including both ISKNV and RSIV, were 49. Although the genome content of SDDV (131 kb) was nearly 20 kb larger than that of ISKNV (111 kb), over 50 additional identified viral proteins in ZH-06/20 virion were far beyond our expectations. Importantly, 113 identified viral proteins were best-matched to ZH-06/20 ORFs, which suggested to some degree that the genome annotation of ZH-06/20 was correct. A refined proteomic identification should be conducted to clarify viral proteins of SDDV in future work. 

To assess the antigenic cross-reaction, both pAbs and mAbs of several well-characterized viral proteins were used. The results showed weak cross-reactions between the major capsid proteins of both the SDDV isolate ZH-06/20 and ISKNV isolate NH060831, using the complete *mcp* gene-based pAbs as the first antibodies. The major capsid protein is the most abundant viral protein in all members of iridoviruses and accounts for about 40% in total virion proteins. Weak cross-reaction at the MCP level suggested the significant antigenic difference between SDDV and ISKNV. Moreover, a weak cross-reaction of ISKNV-VP007, a major envelope protein in ISKNV/RSIV-like megalocytivirus, was also observed using a pAb against ISKNV-VP007 as the first antibody. No cross-reactions were observed using pAbs or mAbs of ISKNV-VP101 and VP023 as the first antibodies to recognize purified ZH-06/20 or ZH-06/20 infected-MFF-1 cells (Figure 6C,D). All of these data suggest that no cross-protection of the RSIV/ISKNV vaccine against SDDV infection is under expectation. Due to the very high antigenic homogeneity, ISKNV-like traditional megalocytivirus has no serotype concept among different isolates [32]. Thus, if SDDV is still listed as a member of the genus *Megalocytivirus* in the future, we propose that these members in megalocytivirus be divided as two serotypes, according to viral antigenicity, namely SDDV serotype and ISKNV serotype, respectively. 

On a histopathological level, basophilic hypertrophied cells (BHC) and intracytoplasmic inclusion body (IB) were observed in SDDV infected tissue from diseased *L. calcarifer* [16]. However, similar histopathological features were not observed in infected yellowfin seabream, although an enlarged spleen and a necrotic liver were also the featured clinical syndromes in YFSBAD fish (Figure 1B). The histopathological features of YFSBAD are also considerably different from those of ISKNV-infected *L. calcarifer.* The featured histopathology of ISKNV-infected *L. calcarifer* are characterized by numerous, normally enlarged cells in all infected tissues, including the spleen, kidney, liver, stomach, and gill [9], but no obvious enlargement cell was observed in SDDV-infected yellowfin seabass tissues (Figure 8 and Figure 9). In ISKNV, ISKNV-ORF023 (VP23) encodes a laminin-like non-structural protein to form virus-mock basement membrane (VMBM) on the surface of infected normally enlarged cells [21]. No VP23 homological gene was found in the SDDV genome, and no cross reactions were observed when pAb and mAb of ISKNV-VP23 were used as the first antibodies to recognize the ZH06/20-infected MFF-1 cell (Figure 6D). It is presumed that the normal enlargement cell may not be the featured histopathology for SDDV-associated diseased. 

Suitable cell lines are essential to virus isolation, diagnostics, and the development of vaccines. For unknown reasons, very few piscine cell lines are actually suitable for effective proliferation of megalocytivirus [20,34]. In a previous study, the mandarin fish fry (MFF-1) cell line was established by our team and showed highly efficient proliferation of both ISKNV and RSIV [20,23]. This study once again showed MFF-1 cell was still a suitable cell line for the study of SDDV. In de Groof’s report, Singaporean SDDV could grow well in seabass kidney (SK) SK21 cells [13]. ECIV, a sister isolate to the SDDV, could grow on EPC, BF-2, CHSE-214, KF-1, and CCB cell lines [18]. In our study, EPC, FHM, and KCF-1 cells also performed a sensitivity test for infection of the adapted ZH-06/20 in MFF-1 cells; however, the propagation efficacies of ZH-06/20 in these cells were considerably lower than we expected.

## 5. Conclusions

An SDDV isolate ZH-06/20 was isolated from YFSBAD yellowfin seabream using the MFF-1 cell line. ZH-06/20 was characterized by cell culture, TEM observation, genomic and proteomic determination, antigenic cross-reaction assessment, artificial infection, histopathology, and IFA assay. All of these data support that SDDV was the causative agent of YFSBA diseases in Zhuhai, South China. To the best of our knowledge, yellowfin seabream is the third confirmed natural host fish species for SDDV infection, and SDDV was isolated for the first time in mainland China. The transboundary virus will be well studied because of its so unsolved issues.

## Data Availability

All data generated or analyzed during this study are included in this published article.

## Supplementary Materials

The following are available online at https://www.mdpi.com/article/10.3390/v13081617/s1, Table S1: Listing of potential expressed ORFs in ZH-06/20, Table S2: Crude virion proteome of ZH-06/20.
