# Supplementary material for: Scale Drop Disease Virus Associated Yellowfin Seabream (Acanthopagrus latus) Ascites Diseases, Zhuhai, Guangdong, Southern China: The First Description
Source: Viruses. 2021 Aug 16;13(8):1617. doi: 10.3390/v13081617 (PMC8402775; doi:10.3390/v13081617)
Supplement: Supplementary file 1 [file viruses-13-01617-s001.zip › Table S1. Listing of potential expressed ORFs in ZH0620.pdf]

Table S1. Listing of potential expressed ORFs in ZH06/20

| ORF | Nucleotide position | MW (kDa) | pI | Conserved domain or signature | Match | | | | Predicted structure and/or function |
|---|---|---|---|---|---|---|---|---|---|
| | | | | | BlastP score | % Identity | Accession no. | Species | |
| ORF001L | 222-1268(1047) | 39.79 | 8.19 | WcaG | 722 | 99.71 | QLI60785.1 | SDDV(3 beta-hydroxysteroid dehydrogenase) | |
| ORF002L | 1272-2216(945) | 36.64 | 8.36 | | 655 | 100.00 | QLI60784.1 | SDDV(hypothetical protein) | ANK |
| | | | | | 55 | 24.27 | QCQ67799.1 | ECIV(ankyrin repeat-containing protein) | |
| ORF003L | 2200-2748(549) | 20.72 | 4.56 | | 374 | 100.00 | QLI60783.1 | SDDV(hypothetical protein) | |
| | | | | | 170 | 53.09 | AMM04443.1 | ISKNV037L | |
| | | | | | 167 | 52.44 | QCQ67800.1 | ECIV045 | |
| ORF004L | 2796-3203(408) | 15.06 | 10.54 | | 269 | 100.00 | QLI60782.1 | SDDV(hypothetical protein) | TM |
| | | | | | 178 | 64.93 | QCQ67797.1 | ECIV(hypothetical protein) | |
| | | | | | 103 | 46.15 | AMM04520.1 | ISKNV131L | |
| ORF005R | 3234-3815(582) | 22.77 | 8.46 | PHA03096 | 404 | 100.00 | QLI60781.1 | SDDV(putative E3 ubiquitin-protein ligase makorin-1) | |
| | | | | | 64 | 37.96 | QCQ67781.1 | ECIV(RING E3 ubiquitin ligase) | |
| | | | | | 61.2 | 31.87 | NP_612234.1 | ISKNV(putative zinc finger protein) | |
| ORF006L | 3800-5143(1344) | 50.50 | 4.69 | Ank_2 | 926 | 100.00 | QLI60780.1 | SDDV(Serine/threonine-protein phosphatase 6 regulatory ankyrin repeat subunit B) | ANK |
| | | | | | 313 | 39.16 | QCQ67799.1 | ECIV(ankyrin repeat-containing protein ) | |
| | | | | | 101 | 24.01 | NP_612341.1 | ISKNV119L | |
| ORF007R | 5207-6670(1464) | 56.32 | 7.37 | | 1009 | 100.00 | QLI60779.1 | SDDV(hypothetical protein) | |
| | | | | | 240 | 32.23 | QCQ67801.1 | ECIV(ankyrin repeat-containing protein) | |
| ORF008R | 6722-12394(5673) | 211.70 | 6.72 | Drf_FH1 Poxvirus_B22(pfam06346) | 3841 | 97.67 | QLI60778.1 | SDDV(hypothetical protein) | signal peptide Pfam:Poxvirus_B22R_C TM |
| | | | | Poxvirus_B22R_C super family(cl16146) | 1825 | 52.11 | QCQ67814.1 | ECIV059 | |

| ORF | Nucleotide position | MW (kDa) | pI | Conserved domain or signature | Match | | | | Predicted structure and/or function |
|---|---|---|---|---|---|---|---|---|---|
| | | | | | BlastP score | % Identity | Accession no. | Species | |
| ORF009R | 12411-15122(2712) | 104.01 | 8.79 | SNF2_N | 1902 | 100.00 | QLI60777.1 | SDDV(putative helicase) | DEXDc Pfam:Helicase_C |
| | | | | DEAD-like_helicase_C | 1324 | 68.28 | QCQ67815.1 | ECIV(DEAD-like helicase) | |
| | | | | | 787 | 45.76 | NP_612285.1 | ISKNV(putative NTPase ) | |
| ORF010R | 15200-15646(447) | 16.54 | 4.26 | | 296 | 99.32 | QLI60776.1 | SDDV(Uncharacterized RING finger protein) | |
| | | | | | 67 | 35.96 | QCQ67816.1 | ECIV(RING E3 ubiquitin ligase) | |
| | | | | | 31 | 36.36 | NP_612287.1 | ISKNV065L | |
| ORF011R | 15683-15997(315) | 12.20 | 7.74 | | 214 | 100.00 | QLI60775.1 | SDDV(Tripartite motif-containing protein) | |
| | | | | | 150 | 61.90 | QCQ67817.1 | ECIV(RING E3 ubiquitin ligase) | |
| | | | | | 88 | 38.61 | AMM04418.1 | ISKNV008L | |
| ORF012R | 16002-16325(324) | 12.38 | 7.30 | | 224 | 100.00 | QLI60774.1 | SDDV(hypothetical protein) | ANK |
| | | | | | 140 | 59.63 | QCQ67818.1 | SDDV(hypothetical protein) | |
| ORF013R | 16331-16888(558) | 21.06 | 7.92 | NIF(pfam03031) | 382 | 100.00 | QLI60773.1 | SDDV(putative CTD phosphatase-like protein) | CPDc |
| | | | | | 282 | 71.12 | QCQ67819.1 | ECIV(NIF/NLI interacting factor) | |
| | | | | | 181 | 53.37 | NP_612227.1 | ISKNV005L | |
| ORF014R | 16893-17351(459) | 17.64 | 7.20 | | 315 | 100.00 | QLI60772.1 | SDDV(hypothetical protein) | TM |
| ORF015R | 17356-18327(972) | 36.78 | 6.35 | serpin(cd00172) | 666 | 100.00 | QLI60771.1 | SDDV(Serine proteinase inhibitor) | SERPIN |
| | | | | | 233 | 38.82 | QCQ67786.1 | ECIV(serpin) | |
| ORF016R | 18406-18783(378) | 14.79 | 8.53 | CTH1 super family(cl34885) | 256 | 99.20 | QLI60770.1 | SDDV(mRNA decay activator protein) | |
| ORF017R | 18983-19531(549) | 20.83 | 7.88 | BI-1-like super family(cl00473) | 366 | 99.45 | QLI60769.1 | SDDV(Protein lifeguard) | |
| ORF018R | 19593-20774(1182) | 45.16 | 6.90 | PTP_DSP_cys super family (cl28904) | 818 | 100.00 | QLI60768.1 | SDDV(hypothetical protein) | |

| ORF | Nucleotide position | MW (kDa) | pI | Conserved domain or signature | Match | | | | Predicted structure and/or function |
|---|---|---|---|---|---|---|---|---|---|
| | | | | | BlastP score | % Identity | Accession no. | Species | |
| | | | | | 418 | 52.79 | QCQ67843.1 | ECIV088 | |
| | | | | | 118 | 25.60 | NP_612304.1 | ISKNV082L | |
| ORF019L | 20802-21314(513) | 19.23 | 4.73 | | 349 | 100.00 | QLI60767.1 | SDDV(hypothetical protein) | |
| | | | | | 161 | 46.34 | QCQ67842.1 | ECIV087 | |
| ORF020L | 21322-21816(495) | 18.67 | 4.14 | | 333 | 100.00 | QLI60766.1 | SDDV(hypothetical protein) | |
| | | | | | 171 | 50.90 | QCQ67841.1 | ECIV086 | |
| ORF021R | 21901-24768(2868) | 111.11 | 6.15 | | 1982 | 99.90 | QLI60765.1 | SDDV(putative structural protein) | |
| | | | | | 1078 | 54.45 | QCQ67840.1 | ECIV085 | |
| | | | | | 578 | 36.30 | NP_612298.1 | ISKNV076L | |
| ORF022R | 24793-25215(423) | 16.15 | 9.03 | Macro_Poa1p-like(cd02901) | 295 | 100.00 | QLI60764.1 | SDDV(ADP-ribose glycohydrolase) | |
| ORF023R | 25228-25791(564) | 21.66 | 7.98 | NK super family(cl17190) | 389 | 99.47 | QLI60763.1 | SDDV(Thymidylate kinase) | Pfam: Thymidylate _ kin |
| | | | | | 180 | 43.39 | QCQ67762.1 | ECIV(thymidylate kinase) | CPDc |
| ORF024R | 25794-27296(1503) | 57.72 | 7.91 | PTP_DSP_cys super family (cl28904) | 1044 | 100.00 | QLI60762.1 | SDDV(mRNA-capping enzyme) | PTPc_DSPc Pfam:mRNA_cap _ enzyme |
| | | | | Adenylation_ mRNA_capping (cd07895) | 600 | 59.80 | QCQ67761.1 | ECIV(mRNA capping enzyme) | |
| | | | | mRNA_cap_C (pfam03919) | 423 | 44.91 | NP_612286.1 | ISKNV(putative RNA guanylytransferase) | |
| ORF025R | 27297-27752(456) | 17.49 | 8.54 | | 203 | 100.00 | QLI60761.1 | SDDV(hypothetical protein) | |
| | | | | | 49 | 39.08 | QCQ67760.1 | ECIV005 | |
| ORF026L | 27800-28402(603) | 23.29 | 9.78 | | 414 | 100.00 | QLI60760.1 | SDDV(hypothetical protein) | |
| | | | | | 193 | 54.46 | QCQ67811.1 | ECIV056 | |
| ORF027R | 28585-29403(819) | 30.60 | 9.27 | RNase_HI_ eukaryote_like (cd09280) | 563 | 100.00 | QLI60759.1 | SDDV(Ribonuclease) | Pfam:RNase_H |

| ORF | Nucleotide position | MW (kDa) | pI | Conserved domain or signature | Match | | | | Predicted structure and/or function |
|---|---|---|---|---|---|---|---|---|---|
| | | | | | BlastP score | % Identity | Accession no. | Species | |
| ORF028L | 29575-30489(915) | 33.89 | 4.77 | Cauli_VI (pfam01693) Ank_2(pfam12796) | 636 | 100.00 | QLI60758.1 | SDDV(hypothetical protein) | ANK |
| | | | | | 378 | 57.43 | QCQ67759.1 | ECIV(ankyrin repeat-containing protein) | |
| | | | | | 120 | 35.03 | NP_612347.1 | ISKNV(putative ankyrin repeat protein) | |
| ORF029R | 30513-31610(1098) | 41.90 | 5.27 | | 751 | 99.73 | QLI60757.1 | SDDV(hypothetical protein) | ANK |
| | | | | | 340 | 49.59 | QCQ67758.1 | ECIV(ankyrin repeat-containing protein) | |
| ORF030L | 31579-32691(1113) | 40.76 | 7.66 | SLC5-6-like_sbd super family(cl00456) | 736 | 100.00 | QLI60756.1 | SDDV(Sodium-coupled neutral amino acid transporter) | Pfam:Aa_trans |
| | | | | | 463 | 62.74 | QCQ67756.1 | ECIV(transmembrane amino acid transporter protein) | |
| | | | | | 199 | 33.88 | NP_612223.1 | ISKNV001L | |
| ORF031L | 32698-33162(465) | 18.23 | 6.34 | RNA_pol_Rpb5_C super family(cl00883) | 322 | 100.00 | QLI60755.1 | SDDV(hypothetical protein) | Pfam:RNA_pol_Rpb5_C |
| | | | | | 167 | 50.31 | QCQ67863.1 | ECIV108 | |
| | | | | | 109 | 34.62 | AMM04413.1 | ISKNV(DNA dependent RNA polymerase subunit H-like protein) | |
| ORF032L | 33152-33649(498) | 18.94 | 7.74 | | 337 | 100.00 | QLI60754.1 | SDDV(hypothetical protein) | |
| | | | | | 197 | 50.00 | QCQ67862.1 | ECIV107 | |
| | | | | | 86 | 28.48 | AMM04500.1 | ISKNV104L | |
| ORF033L | 33661-34179(519) | 19.46 | 6.86 | | 360 | 100.00 | QLI60753.1 | SDDV(hypothetical protein) | |
| | | | | | 262 | 70.76 | QCQ67861.1 | ECIV106 | |
| | | | | | 166 | 49.12 | NP_612323.1 | ISKNV101L | |
| ORF034R | 34178-35062(885) | 33.96 | 8.12 | | 591 | 100.00 | QLI60752.1 | SDDV(hypothetical protein) | |
| | | | | | 231 | 43.20 | QCQ67860.1 | ECIV105 | |

| ORF | Nucleotide position | MW (kDa) | pI | Conserved domain or signature | Match | | | | Predicted structure and/or function |
|---|---|---|---|---|---|---|---|---|---|
| | | | | | BlastP score | % Identity | Accession no. | Species | |
| ORF035R | 35052-38603(3552) | 133.01 | 8.12 | RNAP_largest_subunit_N super family(cl19114) | 2468 | 100.00 | QLI60751.1 | SDDV(DNA-directed RNA polymerase II subunit) | RPOLA_N |
| | | | | RNAP_largest_subunit_C super family(cl29012) | 1892 | 74.70 | QCQ67859.1 | ECIV(DNA-dependent RNA polymerase II largest subunit) | Pfam:RNA_pol_Rpb1_4 |
| | | | | rpoC2_cyan super family(cl37097) | 1375 | 57.99 | NP_612250.1 | ISKNV028L | Pfam:RNA_pol_Rpb1_5 |
| ORF036L | 38654-39304(651) | 25.90 | 8.71 | | 397 | 99.48 | QLI60750.1 | SDDV(hypothetical protein) | |
| | | | | | 346 | 76.50 | QCQ67787.1 | ECIV032 | |
| ORF037L | 39405-40073(669) | 26.70 | 8.46 | | 439 | 100.00 | QLI60749.1 | SDDV(hypothetical protein) | |
| ORF038R | 40073-41038(966) | 37.03 | 7.88 | PTZ00217 super family (cl36527) | 631 | 100.00 | QLI60748.1 | SDDV(Flap endonuclease) | XPGN XPGI HhH2 |
| | | | | | 471 | 74.10 | QCQ67858.1 | ECIV(Flap endonuclease) | |
| | | | | | 320 | 49.84 | NP_612249.1 | ISKNV(putative DNA repair protein) | |
| ORF039L | 41062-42135(1074) | 39.35 | 6.01 | PRK08581 super family (cl35718) | 706 | 99.16 | QLI60747.1 | SDDV(hypothetical protein) | signal peptide TM |
| | | | | | 105 | 42.06 | QCQ67770.1 | ECIV015 | |
| ORF040L | 42153-42731(579) | 21.72 | 9.09 | | 396 | 100.00 | QLI60746.1 | SDDV(hypothetical protein) | SCOP:d1b67a_ |
| | | | | | 244 | 61.26 | QCQ67771.1 | ECIV(short chain dehydrogenase) | |
| | | | | | 118 | 39.10 | AMM04431.1 | ISKNV024R | |
| ORF041L | 42734-45640(2907) | 110.41 | 8.19 | PTZ00166 super family (cl36522) | 2022 | 99.90 | QLI60745.1 | SDDV(DNA polymerase) | POLBc |
| | | | | | 1443 | 70.76 | QCQ67772.1 | ECIV(DNA polymerase family B) | |
| | | | | | 985 | 51.07 | NP_612241.1 | ISKNV(putative DNA polymerase) | |
| ORF042R | 45679-45888(210) | 7.99 | 13.03 | | 112 | 100.00 | QLI60744.1 | SDDV(hypothetical protein) | SCOP:d1e5xa_ |
| | | | | | 44 | 69.70 | NP_612240.1 | ISKNV018L | |
| ORF043L | 45985-46239(255) | 10.18 | 10.39 | | 159 | 100.00 | QLI60743.1 | SDDV(hypothetical protein) | TM |

| ORF | Nucleotide position | MW (kDa) | pI | Conserved domain or signature | Match | | | | Predicted structure and/or function |
|---|---|---|---|---|---|---|---|---|---|
| | | | | | BlastP score | % Identity | Accession no. | Species | |
| ORF044R | 46252-46548(297) | 10.70 | 7.03 | | 199 | 100.00 | QLI60742.1 | SDDV(hypothetical protein) | TM |
| | | | | | 92 | 51.00 | QCQ67773.1 | ECIV018 | |
| | | | | | 39 | 31.37 | AMM04427.1 | ISKNV020L | |
| ORF045R | 46548-47177(630) | 23.69 | 4.51 | | 419 | 99.51 | QLI60741.1 | SDDV(hypothetical protein) | |
| | | | | | 249 | 62.56 | QCQ67774.1 | ECIV019 | |
| | | | | | 103 | 29.13 | NP_612238.1 | ISKNV016L | |
| ORF046L | 47203-48045(843) | 32.89 | 8.24 | | 581 | 100.00 | QLI60740.1 | SDDV(hypothetical protein) | |
| | | | | | 372 | 60.36 | QCQ67785.1 | ECIV(IKI3 family protein) | |
| | | | | | 193 | 37.01 | NP_612237.1 | ISKNV015R | |
| ORF047L | 48048-49913(1866) | 69.00 | 4.36 | Macro_OAADPr_deacetylase (cd02908) | 1280 | 97.95 | QLI60739.1 | SDDV(ADP-ribose glycohydrolase) | internal repeat 1 A1pp |
| | | | | RRM_SF super family (cl17169) | 600 | 49.69 | QCQ67784.1 | ECIV(macro domain-containing protein) | |
| | | | | | 155 | 50.29 | NP_612244.1 | ISKNV022L | |
| ORF048R | 49935-50261(327) | 12.87 | 8.81 | | 231 | 100.00 | QLI60738.1 | SDDV(hypothetical protein) | |
| | | | | | 127 | 57.41 | QCQ67783.1 | ECIV028 | |
| ORF049L | 50268-51875(1608) | 62.25 | 8.90 | | 1125 | 100.00 | QLI60737.1 | SDDV(hypothetical protein) | TM |
| | | | | | 484 | 47.14 | QCQ67782.1 | ECIV(serine/threonine protein kinase) | |
| | | | | | 115 | 25.89 | NP_612235.1 | ISKNV013R | |
| ORF050L | 51811-52155(345) | 13.29 | 8.58 | KilA-N super family (cl29320) | 235 | 100.00 | YP_009163825.1 | SDDV064R | |
| | | | | | 61 | 32.71 | QLI60781.1 | SDDV(putative E3 ubiquitin-protein ligase makorin-1) | |
| | | | | | 130 | 57.69 | QCQ67781.1 | ECIV(RING E3 ubiquitin ligase) | |
| | | | | | 64 | 36.94 | NP_612234.1 | ISKNV(putative zinc finger protein) | |
| ORF051R | 52185-52454(270) | 10.15 | 8.15 | | 177 | 100.00 | QLI60736.1 | SDDV(hypothetical protein) | TM |
| | | | | | 40 | 31.43 | NP_612233.1 | ISKNV011L | |
| ORF052R | 52458-52835(378) | 14.49 | 8.28 | | 263 | 100.00 | QLI60735.1 | SDDV(hypothetical protein) | |
| | | | | | 121 | 41.94 | QCQ67780.1 | ECIV025 | |

| ORF | Nucleotide position | MW (kDa) | pI | Conserved domain or signature | Match | | | | Predicted structure and/or function |
|---|---|---|---|---|---|---|---|---|---|
| | | | | | BlastP score | % Identity | Accession no. | Species | |
| ORF053L | 52842-53111(270) | 10.02 | 9.57 | | 184 | 100.00 | QLI60734.1 | SDDV(hypothetical protein) | |
| | | | | | 51 | 37.84 | QQZ00464.1 | ISKNV(hypothetical protein IJGMMPBP_00011) | |
| ORF054R | 53143-53358(216) | 7.80 | 9.20 | | 143 | 100.00 | QLI60733.1 | SDDV(hypothetical protein) | TM |
| | | | | | 75 | 68.06 | AMM04525.1 | ISKNV012L | |
| ORF055L | 53386-54729(1344) | 50.28 | 7.46 | | 919 | 100.00 | QLI60732.1 | SDDV(hypothetical protein) | |
| | | | | | 298 | 36.48 | QCQ67779.1 | ECIV024 | |
| | | | | | 64 | 21.32 | NP_612230.1 | ISKNV008R | |
| ORF056R | 54791-56341(1551) | 55.78 | 8.28 | L1R_F9L super family (cl28088) | 1056 | 100.00 | QLI60731.1 | SDDV(putative myristoylated protein) | TM |
| | | | | | 728 | 67.64 | QCQ67778.1 | ECIV(myristylated membrane protein) | |
| | | | | | 386 | 43.53 | NP_612229.1 | ISKNV007L | |
| ORF057R | 56345-57706(1362) | 49.97 | 6.77 | Capsid_NCLDV (pfam04451) | 944 | 100.00 | QLI60730.1 | SDDV(Major capsid protein) | Pfam:Capsid_N Pfam:Capsid_NCLDV |
| | | | | Capsid_N super family(cl25189) | 774 | 81.06 | QCQ67796.1 | ECIV MCP | |
| | | | | | 664 | 68.57 | NP_612228.1 | ISKNV(putative MCP) | |
| ORF058L | 57799-58365(567) | 22.00 | 6.08 | | 390 | 100.00 | QLI60729.1 | SDDV(hypothetical protein) | |
| | | | | | 73 | 31.29 | QCQ67838.1 | ECIV083 | |
| ORF059L | 58421-58828(408) | 15.95 | 6.83 | | 219 | 84.44 | QLI60728.1 | SDDV(hypothetical protein) | internal repeat 1 |
| | | | | | 105 | 44.63 | QCQ67798.1 | ECIV043 | |
| ORF060L | 58872-59420(549) | 20.84 | 6.60 | Sar1 (cl00264) | 380 | 100.00 | QLI60727.1 | SDDV(GTP-binding protein) | SAR |
| ORF061L | 59449-60273(825) | 31.79 | 7.73 | PKc_like super family (cl21453) | 568 | 100.00 | QLI60726.1 | SDDV(Serine/threonine-protein kinase/endoribonuclease) | STYKc |
| ORF062L | 60342-61256(915) | 35.69 | 6.96 | | 635 | 99.34 | QLI60725.1 | SDDV(hypothetical protein) | |
| | | | | | 251 | 45.45 | QCQ67808.1 | ECIV053 | |
| | | | | | 200 | 36.96 | NP_612294.1 | ISKNV072R | |
| ORF063L | 61261-61908(648) | 24.85 | 8.13 | | 442 | 100.00 | QLI60724.1 | SDDV(hypothetical protein) | |
| | | | | | 72 | 29.30 | QCQ67792.1 | ECIV037 | |

| ORF | Nucleotide position | MW (kDa) | pI | Conserved domain or signature | Match | | | | Predicted structure and/or function |
|---|---|---|---|---|---|---|---|---|---|
| | | | | | BlastP score | % Identity | Accession no. | Species | |
| ORF064L | 61974-62939(966) | 36.92 | 4.92 | Ferritin_like super family (cl00264) | 669 | 100.00 | QLI60723.1 | SDDV(Ribonucleoside-diphosphate reductase subunit) | |
| | | | | | 499 | 75.45 | QCQ67809.1 | ECIV(ribonucleotide reductase beta subunit) | |
| | | | | | 350 | 57.19 | NP_612246.1 | ISKNV(putative ribonucleotide reductase small subunit) | |
| ORF065L | 62973-63602(630) | 25.10 | 9.58 | | 430 | 99.04 | QLI60722.1 | SDDV(hypothetical protein) | |
| ORF066L | 63649-64422(774) | 29.62 | 6.34 | | 531 | 100.00 | QLI60721.1 | SDDV(hypothetical protein) | |
| | | | | | 95 | 27.11 | QCQ67776.1 | ECIV021 | |
| ORF067L | 64434-65132(699) | 25.96 | 7.59 | TNFRSF super family (cl22855) | 471 | 100.00 | QLI60720.1 | SDDV(Tumor necrosis factor receptor superfamily member) | TNFR TM |
| ORF068L | 65180-65887(708) | 26.93 | 6.16 | PTZ00164 super family (cl36520) | 494 | 99.57 | QLI60719.1 | SDDV(Thymidylate synthase) | Pfam:Thymidylat _synt |
| ORF069L | 66036-66824(789) | 30.61 | 7.83 | EEP super family (cl00490) | 546 | 100.00 | QLI60718.1 | SDDV(Deoxyribonuclease-1) | DNaseIc |
| | | | | | 284 | 53.88 | QCQ67810.1 | ECIV(deoxyribonuclease 1) | |
| ORF070R | 66867-67409(543) | 20.98 | 4.78 | | 378 | 100.00 | QLI60717.1 | SDDV(hypothetical protein) | signal peptide |
| | | | | | 117 | 36.31 | QCQ67807.1 | ECIV052 | |
| ORF071L | 67434-68381(948) | 35.82 | 4.69 | Serpin (pfam00079) | 659 | 100.00 | QLI60716.1 | SDDV(Serine proteinase inhibitor) | SERPIN |
| | | | | | 247 | 42.45 | QCQ67812.1 | ECIV(serpin) | |
| ORF072L | 68456-69364(909) | 34.89 | 3.58 | | 597 | 99.67 | QLI60715.1 | SDDV(hypothetical protein) | |
| | | | | | 130 | 36.74 | QCQ67844.1 | ECIV089 | |
| ORF073L | 69569-72301(2733) | 104.84 | 5.22 | primase_Cterm super family (cl36938) | 1907 | 100.00 | QLI60713.1 | SDDV(putative helicase) | |
| | | | | D5_N (pfam08706) | 1264 | 65.51 | QCQ67845.1 | ECIV(D5 family NTPase) | |

| ORF | Nucleotide position | MW (kDa) | pI | Conserved domain or signature | Match | | | | Predicted structure and/or function |
|---|---|---|---|---|---|---|---|---|---|
| | | | | | BlastP score | % Identity | Accession no. | Species | |
| ORF074R | 72308-73051(744) | 27.86 | 7.84 | PriCT_2 (pfam08707) | 940 | 51.24 | NP_612331.1 | ISKNV109L | |
| | | | | | 511 | 100.00 | QLI60712.1 | SDDV(hypothetical protein) | |
| | | | | | 456 | 87.45 | QCQ67846.1 | ECIV(proliferating cell nuclear antigen) | |
| | | | | | 255 | 54.47 | NP_612334.1 | ISKNV112R | |
| ORF075L | 73094-73612(519) | 20.26 | 6.14 | | 352 | 100.00 | QLI60711.1 | SDDV(hypothetical protein) | |
| | | | | | 304 | 83.72 | QCQ67848.1 | ECIV093 | |
| | | | | | 187 | 60.99 | AMM04511.1 | ISKNV119L | |
| ORF076L | 73623-76238(2616) | 100.23 | 7.56 | | 1835 | 100.00 | QLI60710.1 | SDDV(putative kinase) | SCOP:d1kq4a_ |
| | | | | | 940 | 52.11 | QCQ67849.1 | ECIV(tyrosine kinase) | Blast:CAP10 |
| | | | | | 399 | 30.52 | NP_612336.1 | ISKNV114L | SCOP:d1rpxa_ d1ks9a1 d1b6cb_ |
| ORF077R | 76324-77367(1044) | 40.04 | 6.90 | | 729 | 100.00 | QLI60709.1 | SDDV(Immediate-early protein ICP-46 homolog) | |
| | | | | | 444 | 60.34 | QCQ67850.1 | ECIV(immediate early protein ICP-46) | |
| | | | | | 235 | 40.29 | NP_612337.1 | ISKNV115R | |
| ORF078R | 77374-78111(738) | 27.97 | 7.36 | AAA (smart00382) | 511 | 100.00 | QLI60708.1 | SDDV(hypothetical protein) | SCOP:d1e9ra_ |
| | | | | | 466 | 100.00 | QKR72226.1 | SDDV(ATPase) | |
| | | | | | 448 | 88.70 | QCQ67851.1 | ECIV(ATPase) | |
| | | | | | 372 | 73.39 | NP_612345.1 | ISKNV(putative adenosine triphosphatase) | |
| ORF079L | 78140-78832(693) | 25.97 | 6.73 | DUF2738 super family (cl12506) | 478 | 100.00 | QLI60707.1 | SDDV(hypothetical protein) | Pfam:DUF2738 |
| | | | | | 356 | 70.61 | QCQ67853.1 | ECIV098 | |
| | | | | | 139 | 37.18 | NP_612340.1 | ISKNV118L | |
| ORF080R | 78865-79674(810) | 30.67 | 5.85 | | 552 | 99.63 | QLI60706.1 | SDDV(hypothetical protein) | coiled coil |
| | | | | | 310 | 55.51 | QCQ67855.1 | ECIV100 | |
| | | | | | 167 | 38.55 | NP_612318.1 | ISKNV096L | |

| ORF | Nucleotide position | MW (kDa) | pI | Conserved domain or signature | Match | | | | Predicted structure and/or function |
|---|---|---|---|---|---|---|---|---|---|
| | | | | | BlastP score | % Identity | Accession no. | Species | |
| ORF081R | 79690-80826(1137) | 43.35 | 5.93 | | 789 | 100.00 | QLI60705.1 | SDDV(hypothetical protein) | coiled coil |
| | | | | | 360 | 47.56 | QCQ67856.1 | ECIV101 | |
| | | | | | 33 | 35.71 | NP_612317.1 | ISKNV095L | |
| ORF082R | 80823-81773(951) | 34.92 | 9.65 | | 638 | 100.00 | QLI60704.1 | SDDV(hypothetical protein) | |
| | | | | | 300 | 49.85 | QCQ67805.1 | ECIV(proteasome accessory factor) | |
| | | | | | 94 | 32.01 | NP_612315.1 | ISKNV093L | |
| ORF083L | 81795-81974(180) | 6.99 | 4.02 | | 121 | 100.00 | QLI60703.1 | SDDV(hypothetical protein) | TM |
| | | | | | 41 | 38.00 | NP_612314.1 | ISKNV092R | |
| ORF084R | 82002-83072(1071) | 39.81 | 8.51 | | 708 | 97.75 | QLI60702.1 | SDDV(putative membrane protein) | Pfam:Pox_G9-A16 TM |
| | | | | | 462 | 66.01 | QCQ67806.1 | ECIV051 | |
| | | | | | 287 | 49.09 | AMM04534.1 | ISKNV(myristylated membrane protein) | |
| ORF085R | 83085-83597(513) | 18.91 | 4.36 | | 349 | 99.41 | QLI60701.1 | SDDV(hypothetical protein) | signal peptide |
| | | | | | 187 | 56.10 | QCQ67807.1 | ECIV052 | |
| | | | | | 93 | 33.99 | AMM04471.1 | ISKNV066L | |
| ORF086L | 83594-85297(1704) | 65.86 | 7.20 | | 1168 | 100.00 | QLI60700.1 | SDDV(hypothetical protein) | |
| | | | | | 664 | 57.42 | QCQ67791.1 | ECIV036 | |
| | | | | | 219 | 29.36 | NP_612310.1 | ISKNV088R | |
| ORF087R | 85296-85736(441) | 16.69 | 8.93 | SAP (pfam02037) | 297 | 100.00 | QLI60699.1 | SDDV(putative SAP domain-containing protein) | SAP |
| | | | | | 199 | 64.83 | QCQ67789.1 | ECIV(SAP domain-containing protein) | |
| | | | | | 121 | 43.17 | AMM04494.1 | ISKNV(SAP domain-containing ribonucleoprotein) | |
| ORF088L | 85753-87717(1965) | 72.42 | 6.75 | SpoVK (COG0464) CDC48_N (smart01073) AAA (pfam00004) | 1343 | 100.00 | QLI60698.1 | SDDV(Vesicle-fusing ATPase) | AAA |

| ORF | Nucleotide position | MW (kDa) | pI | Conserved domain or signature | Match | | | | Predicted structure and/or function |
|---|---|---|---|---|---|---|---|---|---|
| | | | | | BlastP score | % Identity | Accession no. | Species | |
| ORF089L | 87765-88538(774) | 29.38 | 7.90 | RIBOc super family (cl00258) | 533 | 100.00 | QLI60697.1 | SDDV(putative ribonuclease) | RIBOc |
| | | | | Rnc super family (cl33956) | 377 | 67.70 | QCQ67765.1 | ECIV(ribonuclease III) | |
| | | | | | 273 | 48.03 | NP_612309.1 | ISKNV(putative ribonuclease ) | |
| ORF090L | 88541-88981(441) | 17.19 | 8.75 | | 304 | 100.00 | QLI60696.1 | SDDV(hypothetical protein) | |
| | | | | | 149 | 52.90 | NP_612308.1 | ISKNV086L | |
| ORF091L | 89018-89578(561) | 21.75 | 6.10 | | 384 | 98.92 | QLI60695.1 | SDDV(hypothetical protein) | |
| | | | | | 118 | 39.66 | QCQ67767.1 | ECIV012 | |
| | | | | | 47 | 26.04 | NP_612343.1 | ISKNV121R | |
| ORF092R | 89730-90110(381) | 14.18 | 7.41 | | 256 | 100.00 | QLI60694.1 | SDDV(hypothetical protein) | |
| | | | | | 67 | 25.60 | QCQ67768.1 | ECIV013 | |
| ORF093R | 90112-90489(378) | 14.22 | 7.24 | | 261 | 100.00 | QLI60693.1 | SDDV(hypothetical protein) | |
| | | | | | 103 | 35.77 | QCQ67768.1 | ECIV013 | |
| ORF094R | 90521-90808(288) | 10.82 | 7.95 | | 197 | 100.00 | QLI60692.1 | SDDV(hypothetical protein) | |
| | | | | | 132 | 68.42 | QCQ67769.1 | ECIV014 | |
| ORF095R | 91126-94077(2952) | 112.79 | 5.73 | PRK12678 super family (cl36163) | 1997 | 100.00 | QLI60691.1 | SDDV(hypothetical protein) | |
| | | | | | 426 | 39.10 | QCQ67836.1 | ECIV081 | |
| | | | | | 172 | 28.93 | NP_612284.1 | ISKNV062L | |
| ORF096R | 94082-94921(840) | 32.67 | 9.35 | Pox_VLTF3 super family (cl37538) | 582 | 100.00 | QLI60690.1 | SDDV(putative transcription factor) | Pfam:Pox_VLTF3 |
| | | | | | 427 | 71.58 | QCQ67834.1 | ECIV(transcription factor) | |
| | | | | | 214 | 41.64 | NP_612283.1 | ISKNV061L | |
| ORF097R | 94900-95196(297) | 11.25 | 6.08 | | 201 | 98.98 | QLI60689.1 | SDDV(hypothetical protein) | |
| | | | | | 137 | 64.44 | QCQ67833.1 | ECIV078 | |
| | | | | | 67 | 40.45 | NP_612279.1 | ISKNV057L | |

| ORF | Nucleotide position | MW (kDa) | pI | Conserved domain or signature | Match | | | | Predicted structure and/or function |
|---|---|---|---|---|---|---|---|---|---|
| | | | | | BlastP score | % Identity | Accession no. | Species | |
| ORF098R | 95200-95835(636) | 23.92 | 5.01 | | 421 | 100.00 | QLI60688.1 | SDDV(hypothetical protein) | |
| | | | | | 342 | 78.77 | QCQ67832.1 | ECIV077 | |
| | | | | | 252 | 57.41 | NP_612278.1 | ISKNV056L | |
| ORF099R | 95841-96659(819) | 30.36 | 10.28 | 2C_adapt(pfam08793) | 547 | 99.63 | QLI60687.1 | SDDV(putative serine/threonine-protein kinase) | Pfam:2C_adapt |
| | | | | 2C_adapt super family (cl07414) | 235 | 48.38 | QCQ67831.1 | ECIV(2-cysteine adaptor domain-containing protein) | |
| | | | | | 172 | 42.44 | NP_612277.1 | ISKNV055L | |
| ORF100R | 96687-97565(879) | 33.55 | 9.91 | 2C_adapt(pfam08793) | 612 | 100.00 | QLI60686.1 | SDDV(hypothetical protein) | Pfam:2C_adapt |
| | | | | 2C_adapt super family (cl07414) | 313 | 51.10 | QCQ67830.1 | ECIV(2-cysteine adaptor domain-containing protein) | |
| | | | | | 126 | 31.72 | NP_612276.1 | ISKNV054L | |
| ORF101R | 97572-97778(207) | 7.50 | 9.08 | | 138 | 100.00 | QLI60685.1 | SDDV(hypothetical protein) | TM |
| | | | | | 75 | 59.32 | QQZ00505.1 | ISKNV(hypothetical protein IJGMMPBP_00052) | |
| ORF102R | 97820-98215(396) | 14.98 | 7.83 | Bcl-2_like super family (cl02575) | 270 | 98.47 | QLI60684.1 | SDDV(hypothetical protein) | SCOP:d1k3ka_ |
| | | | | | 109 | 39.10 | QCQ67829.1 | ECIV(Bcl-2 apoptosis regulator protein) | |
| ORF103L | 98181-98432(252) | 9.62 | 6.42 | | 167 | 100.00 | QLI60683.1 | SDDV(hypothetical protein) | |
| ORF104L | 98462-98839(378) | 14.71 | 6.45 | W2 super family (cl17013) | 250 | 100.00 | QLI60682.1 | SDDV(Eukaryotic translation initiation factor) | Blast:eIF5C |
| ORF105L | 98845-99198(354) | 12.99 | 8.70 | PDGF super family (cl00116) | 241 | 100.00 | QLI60681.1 | SDDV(Vascular endothelial growth factor A) | signal peptide PDGF |
| | | | | | 84 | 41.18 | NP_612270.1 | ISKNV048R | |
| ORF106L | 99211-99564(354) | 13.39 | 9.53 | | 239 | 100.00 | QLI60680.1 | SDDV(hypothetical protein) | |
| | | | | | 155 | 63.48 | QCQ67827.1 | ECIV072 | |
| | | | | | 65 | 46.67 | NP_612269.1 | ISKNV047R | |
| ORF107L | 99545-99730 (186) | 7.12 | 9.42 | | 124 | 100.00 | QLI60679.1 | SDDV(hypothetical protein) | TM SCOP:d1fnna1 |

| ORF | Nucleotide position | MW (kDa) | pI | Conserved domain or signature | Match | | | | Predicted structure and/or function |
|---|---|---|---|---|---|---|---|---|---|
| | | | | | BlastP score | % Identity | Accession no. | Species | |
| ORF108R | 99806-100189(384) | 14.83 | 5.29 | | 250 | 100.00 | QLI60678.1 | SDDV(hypothetical protein) | |
| ORF109L | 100139-100522(384) | 14.46 | 6.10 | | 267 | 100.00 | QLI60677.1 | SDDV(hypothetical protein) | |
| ORF110R | 100571-101266(696) | 26.43 | 9.17 | Dcm (COG0270) | 486 | 100.00 | QLI60676.1 | SDDV(putative DNA (cytosine-5)-methyltransferase) | SCOP:d1dcta_ |
| | | | | | 310 | 65.22 | QCQ67826.1 | ECIV(cytosine DNA methyltransferase) | |
| | | | | | 288 | 61.78 | NP_612268.1 | ISKNV(putative cytosine DNA methyltransferase) | |
| ORF111R | 101235-102863(1629) | 62.69 | 6.82 | | 1100 | 99.81 | QLI60675.1 | SDDV(hypothetical protein) | coiled coil |
| | | | | | 213 | 29.07 | QCQ67825.1 | ECIV070 | |
| ORF112R | 102865-104310(1446) | 54.28 | 4.97 | | 984 | 100.00 | QLI60674.1 | SDDV(hypothetical protein) | |
| | | | | | 248 | 33.13 | QCQ67824.1 | ECIV069 | |
| ORF113R | 104316-104678(363) | 13.71 | 8.93 | Evr1_Alr (pfam04777) | 254 | 100.00 | QLI60673.1 | SDDV(putative FAD-linked sulfhydryl oxidase) | Pfam:Evr1_Alr |
| | | | | | 204 | 81.03 | QCQ67823.1 | ECIV(Ervl/Alr family protein) | |
| | | | | | 154 | 59.48 | NP_612265.1 | ISKNV043L | |
| ORF114R | 104671-104829(159) | 6.42 | 9.57 | | 94 | 100.00 | QLI60672.1 | SDDV(hypothetical protein) | |
| ORF115L | 104846-105946(1101) | 42.10 | 4.61 | | 740 | 100.00 | QLI60671.1 | SDDV(hypothetical protein) | |
| | | | | | 190 | 31.27 | QCQ67822.1 | ECIV067 | |
| ORF116R | 105970-107544(1575) | 61.28 | 4.87 | Lipoprotein_7 super family (cl25543) | 1069 | 99.81 | QLI60670.1 | SDDV(hypothetical protein) | |
| | | | | | 122 | 23.11 | QCQ67821.1 | ECIV(chromosome segregation protein) | |
| ORF117R | 107554-108966(1413) | 53.65 | 4.78 | | 959 | 100.00 | QLI60669.1 | SDDV(hypothetical protein) | |
| ORF118L | 108956-110041(1086) | 42.07 | 5.78 | | 729 | 100.00 | QLI60668.1 | SDDV(hypothetical protein) | |
| ORF119R | 110065-111045(981) | 37.99 | 4.78 | | 654 | 99.39 | QLI60667.1 | SDDV(hypothetical protein) | |
| ORF120L | 111078-112511(1434) | 55.16 | 4.96 | | 936 | 100.00 | QLI60666.1 | SDDV(hypothetical protein) | |
| ORF121L | 112471-114024(1554) | 59.13 | 5.45 | SHE3 super family (cl25925) | 1063 | 99.81 | QLI60665.1 | SDDV(hypothetical protein) | |

| ORF | Nucleotide position | MW (kDa) | pI | Conserved domain or signature | Match | | | | Predicted structure and/or function |
|---|---|---|---|---|---|---|---|---|---|
| | | | | | BlastP score | % Identity | Accession no. | Species | |
| ORF122L | 114040-115983(1944) | 75.06 | 5.88 | | 1343 | 99.54 | QLI60664.1 | SDDV(Serine/threonine-protein phosphatase 6 regulatory ankyrin repeat subunit) | ANK |
| | | | | | 496 | 41.79 | QCQ67793.1 | ECIV038 | |
| ORF123L | 116031-117680(1650) | 63.40 | 4.69 | | 1131 | 99.82 | QLI60663.1 | SDDV(hypothetical protein) | ANK |
| | | | | | 412 | 41.22 | QCQ67794.1 | ECIV039 | |
| ORF124L | 117795-117995(201) | 7.72 | 8.71 | TFIIS_C (pfam01096) | 137 | 100.00 | QLI60662.1 | SDDV(DNA-directed RNA polymerase III subunit) | ZnF_C2C2 |
| | | | | | 91 | 66.67 | QCQ67795.1 | ECIV040 | |
| | | | | | 56 | 41.54 | NP_612251.1 | ISKNV029L | |
| ORF125R | 117994-118662(669) | 26.12 | 5.02 | NK super family (cl17190) | 398 | 100.00 | QLI60661.1 | SDDV(putative deoxynucleoside kinase) | Pfam:Dnk |
| | | | | | 163 | 42.78 | NP_612254.1 | ISKNV(putative thymidine kinase) | |
| ORF126L | 118659-119939(1281) | 47.65 | 5.08 | Ank_2 super family (cl39094) | 876 | 100.00 | QLI60660.1 | SDDV(putative ankyrin repeat protein) | ANK |
| | | | | | 156 | 28.95 | QLI60652.1 | SDDV(pI332:I347utative ankyrin repeat protein) | |
| | | | | | 110 | 25.00 | QCQ67847.1 | ECIV(ankyrin repeat-containing protein) | |
| | | | | | 95 | 27.96 | NP_612299.1 | ISKNV(putative ankyrin repeat protein ) | |
| ORF127R | 120008-120721(714) | 26.90 | 7.95 | TNFRSF super family (cl22855) | 494 | 100.00 | QLI60659.1 | SDDV(Tumor necrosis factor receptor superfamily) | signal peptide TNFR TM |
| ORF128R | 120766-123894(3129) | 117.22 | 8.44 | RNA_pol_B_RPB2(cd00653) | 2190 | 100.00 | QLI60658.1 | SDDV(DNA-directed RNA polymerase II subunit) | Pfam:RNA_pol_Rpb2_1 Pfam:RNA_pol_Rpb2_3 |
| | | | | RNA_pol_Rpb2_4(pfam04566) | 1788 | 79.43 | QCQ67777.1 | ECIV(DNA-dependent RNA polymerase II second largest subunit) | Pfam:RNA_pol_Rpb2_6 |
| | | | | | 1283 | 59.60 | NP_612256.1 | ISKNV(putative DNA-directed RNA polymerase II) | Pfam:RNA_pol_Rpb2_7 |

| ORF | Nucleotide position | MW (kDa) | pI | Conserved domain or signature | Match | | | | Predicted structure and/or function |
|---|---|---|---|---|---|---|---|---|---|
| | | | | | BlastP score | % Identity | Accession no. | Species | |
| ORF129R | 123905-125599(1695) | 65.04 | 4.58 | Ank_2 super family(cl39094) | 1154 | 99.65 | QLI60657.1 | SDDV(hypothetical protein) | ANK |
| | | | | | 233 | 29.04 | QCQ67793.1 | ECIV038 | |
| ORF130R | 125645-126583(939) | 35.86 | 8.23 | zf-C3HC4_3 (pfam13920) | 653 | 100.00 | QLI60656.1 | SDDV(hypothetical protein) | RING |
| ORF131L | 126580-126930(351) | 13.71 | 5.70 | | 238 | 100.00 | QLI60655.1 | SDDV(hypothetical protein) | |
| | | | | | 159 | 66.09 | QCQ67775.1 | ECIV020 | |
| | | | | | 71 | 42.74 | AMM04489.1 | ISKNV089R | |
| ORF132R | 126957-127742(786) | 30.55 | 7.04 | | 546 | 100.00 | QLI60654.1 | SDDV(hypothetical protein) | |
| | | | | | 184 | 40.30 | QCQ67776.1 | ECIV021 | |
| ORF133R | 127832-128575(744) | 28.63 | 8.48 | | 520 | 100.00 | QLI60653.1 | SDDV(hypothetical protein) | |
| | | | | | 101 | 29.58 | QCQ67776.1 | ECIV021 | |
| ORF134L | 128559-129980(1422) | 54.21 | 4.99 | PHA03095 super family (cl33707) | 967 | 99.79 | QLI60652.1 | SDDV(putative ankyrin repeat protein) | ANK |
| | | | | Ank_2 super family (cl39094) | 266 | 37.19 | QCQ67857.1 | ECIV(ankyrin repeat-containing protein) | |
| | | | | | 91 | 23.88 | NP_612299.1 | ISKNV(putative ankyrin repeat protein) | |
| ORF135L | 129996-130907(912) | 34.03 | 4.80 | Ank_2 super family(cl39094) | 625 | 99.67 | QLI60651.1 | SDDV(hypothetical protein) | ANK |
| | | | | | 180 | 36.11 | QCQ67799.1 | ECIV(ankyrin repeat-containing protein) | SCOP:d1awcb_ |
