# Supplementary material for: Scale Drop Disease Virus Associated Yellowfin Seabream (Acanthopagrus latus) Ascites Diseases, Zhuhai, Guangdong, Southern China: The First Description
Source: Viruses. 2021 Aug 16;13(8):1617. doi: 10.3390/v13081617 (PMC8402775; doi:10.3390/v13081617)
Supplement: Supplementary file 1 [file viruses-13-01617-s001.zip › Table S2. Virion proteome data of ZH0620.pdf]

Table S2. Crude virion proteome of ZH06/20

	Reference	YFSBV ORF	PepCount	Unique PepCount	CoverPercent	MW(KDa)	PI
1	QLI60691.1	ORF095R	127	62	69.55%	109.91	5.61
2	QLI60777.1	ORF009R	69	55	53.27%	103.88	8.99
3	QLI60765.1	ORF021R	75	45	56.16%	113.38	5.91
4	QLI60700.1	ORF086L	67	38	57.89%	64.80	6.91
5	QLI60663.1	ORF123L	86	35	63.39%	63.46	4.87
6	QLI60665.1	ORF121L	45	33	67.70%	59.11	5.37
7	QLI60713.1	ORF073L	36	28	32.53%	104.84	5.27
8	QLI60687.1	ORF099R	85	26	83.52%	30.46	10.26
9	QLI60664.1	ORF122L	46	26	39.26%	75.08	5.67
10	QLI60778.1	ORF008R	28	26	13.32%	207.16	6.45
11	QLI60710.1	ORF076L	27	25	36.17%	100.23	7.68
12	QLI60730.1	ORF057R	102	24	73.73%	49.97	6.46
13	QLI60686.1	ORF100R	68	24	72.60%	33.55	9.91
14	QLI60739.1	ORF047L	48	23	48.11%	70.23	4.45
15	QLI60731.1	ORF056R	45	21	40.50%	55.78	8.40
16	QLI60657.1	ORF129R	28	20	34.75%	64.98	4.70
17	QLI60702.1	ORF084R	26	18	39.94%	38.96	8.72
18	QLI60666.1	ORF120L	23	18	33.41%	52.80	4.94
19	QLI60705.1	ORF081R	30	17	58.47%	43.35	5.75
20	QLI60675.1	ORF111R	23	17	39.44%	61.83	6.48
21	QLI60782.1	ORF004L	83	16	70.37%	14.94	10.52
22	QLI60709.1	ORF077R	23	16	52.74%	40.04	6.60
23	QLI60654.1	ORF132R	20	16	57.09%	30.55	6.71
24	QLI60667.1	ORF119R	18	16	48.47%	37.98	4.96
25	QLI60771.1	ORF015R	20	15	44.58%	36.65	6.11
26	QLI60737.1	ORF049L	19	15	28.60%	62.12	9.07
27	QLI60752.1	ORF034R	18	15	60.28%	32.94	7.20
28	QLI60745.1	ORF041L	17	15	17.46%	110.30	8.50
29	QLI60711.1	ORF075L	24	14	51.16%	20.26	5.96
30	QLI60652.1	ORF134L	21	14	30.02%	54.24	5.13
31	QLI60748.1	ORF038R	19	14	44.92%	34.93	7.62
32	QLI60670.1	ORF116R	18	14	30.34%	61.28	4.93
33	QLI60749.1	ORF037L	19	13	63.68%	25.26	8.48
34	QLI60688.1	ORF098R	18	13	65.85%	23.20	4.91
35	QLI60658.1	ORF128R	14	13	13.92%	117.22	8.76
36	QLI60697.1	ORF089L	13	13	40.08%	29.38	8.06
37	QLI60698.1	ORF088L	13	13	20.18%	72.42	6.48
38	QLI60660.1	ORF126L	18	12	33.57%	47.65	5.11
39	QLI60669.1	ORF117R	17	12	27.87%	53.65	4.86
40	QLI60780.1	ORF006L	17	12	34.45%	50.50	4.82
41	QLI60671.1	ORF115L	14	12	45.36%	42.10	4.75
42	QLI60751.1	ORF035R	13	12	9.72%	132.88	8.44
43	QLI60753.1	ORF033L	25	11	80.81%	19.33	6.52
44	QLI60758.1	ORF028L	19	11	40.13%	33.77	4.90

	Reference	YFSBV ORF	PepCount	Unique PepCount	CoverPercent	MW(KDa)	PI
45	QLI60708.1	ORF078R	16	11	53.88%	27.97	7.04
46	QLI60757.1	ORF029R	15	11	36.16%	41.74	5.34
47	QLI60674.1	ORF112R	12	11	31.19%	54.28	5.07
48	QLI60763.1	ORF023R	12	11	65.24%	21.49	8.52
49	QLI60746.1	ORF040L	25	10	58.85%	21.59	9.15
50	QLI60735.1	ORF052R	22	10	64.80%	14.49	8.43
51	QLI60651.1	ORF006L	15	10	32.01%	33.96	4.95
52	QLI60785.1	ORF001L	11	10	31.03%	39.70	8.49
53	QLI60721.1	ORF067L	15	9	50.58%	29.62	6.06
54	QLI60734.1	ORF053L	15	9	79.78%	10.02	9.58
55	QLI60717.1	ORF070R	10	8	49.44%	20.98	4.90
56	QLI60740.1	ORF046L	10	8	28.67%	32.63	8.50
57	QLI60768.1	ORF018R	9	8	17.56%	45.03	6.65
58	QLI60701.1	ORF085R	12	7	46.47%	18.91	4.48
59	QLI60706.1	ORF080R	9	7	24.54%	30.64	5.71
60	QLI60707.1	ORF079L	7	7	28.26%	25.97	6.44
61	QLI60762.1	ORF024R	7	7	12.20%	57.72	8.21
62	QLI60744.1	ORF042R	22	6	37.68%	7.99	12.93
63	QLI60742.1	ORF044R	19	6	48.98%	10.57	6.54
64	QLI60712.1	ORF074R	9	6	23.48%	27.86	7.73
65	QLI60736.1	ORF051R	9	6	44.94%	10.02	7.86
66	QLI60715.1	ORF072L	8	6	19.21%	34.79	3.79
67	QLI60668.1	ORF118L	7	6	19.94%	42.07	5.64
68	QLI60704.1	ORF082R	7	6	24.68%	34.92	9.63
69	QLI60767.1	ORF019L	7	6	38.24%	19.10	4.87
70	QLI60773.1	ORF013R	7	6	29.73%	20.93	7.79
71	QLI60766.1	ORF020L	10	5	39.02%	18.54	4.29
72	QLI60764.1	ORF022R	9	5	32.14%	16.02	9.22
73	QLI60655.1	ORF131L	5	5	55.17%	13.71	5.52
74	QLI60676.1	ORF109R	5	5	31.17%	26.43	9.25
75	QLI60781.1	ORF005R	5	5	21.76%	22.64	8.83
76	QLI60755.1	ORF031L	5	4	22.73%	18.10	6.09
77	QLI60659.1	ORF127R	4	4	23.63%	26.90	8.31
78	QLI60683.1	ORF103L	4	4	60.24%	9.62	6.06
79	QLI60693.1	ORF093R	4	4	36.00%	14.22	6.88
80	QLI60718.1	ORF069L	4	4	16.03%	30.61	8.00
81	QLI60741.1	ORF045R	4	4	25.49%	22.99	4.63
82	QLI60743.1	ORF043L	10	3	25.97%	9.18	10.21
83	QLI60703.1	ORF083L	6	3	62.71%	6.99	4.19
84	QLI60656.1	ORF130R	4	3	10.90%	35.86	8.56
85	QLI60681.1	ORF105L	4	3	35.04%	12.99	9.02
86	QLI60684.1	ORF102R	4	3	22.14%	14.95	7.49
87	QLI60724.1	ORF063L	4	3	16.74%	24.85	8.36
88	QLI60678.1	ORF108R	3	3	36.07%	14.25	5.55
89	QLI60696.1	ORF090L	3	3	30.14%	17.19	8.95
90	QLI60732.1	ORF055L	3	3	7.61%	50.28	7.66

	Reference	YFSBV ORF	PepCount	Unique PepCount	CoverPercent	MW(KDa)	PI
91	QLI60738.1	ORF048R	3	3	29.63%	12.75	8.91
92	QLI60774.1	ORF012R	3	3	36.45%	12.25	6.89
93	QLI60783.1	ORF003L	3	3	22.53%	20.72	4.69
94	QLI60733.1	ORF054R	3	2	11.27%	7.80	9.21
95	QLI60653.1	ORF133R	2	2	13.20%	28.96	8.66
96	QLI60699.1	ORF087R	2	2	9.59%	16.69	9.00
97	QLI60726.1	ORF061L	2	2	8.76%	31.79	7.65
98	QLI60747.1	ORF039L	2	2	5.60%	39.24	5.66
99	QLI60761.1	ORF025R	2	2	20.62%	11.03	9.00
100	QLI60692.1	ORF094R	2	1	12.70%	14.41	9.38
101	QLI60661.1	ORF125R	1	1	2.63%	22.48	5.08
102	QLI60679.1	ORF107R	1	1	19.67%	7.12	9.51
103	QLI60680.1	ORF106L	1	1	8.55%	13.39	9.52
104	QLI60685.1	ORF101R	1	1	14.71%	7.50	9.18
105	QLI60689.1	ORF097R	1	1	11.22%	11.23	5.83
106	QLI60690.1	ORF096R	1	1	2.51%	32.67	9.37
107	QLI60695.1	ORF091L	1	1	5.38%	21.81	5.84
108	QLI60719.1	ORF068L	1	1	2.79%	32.88	6.54
109	QLI60720.1	ORF067L	1	1	4.74%	25.96	7.78
110	QLI60723.1	ORF064L	1	1	3.43%	36.92	4.99
111	QLI60725.1	ORF062L	1	1	1.64%	35.70	6.49
112	QLI60728.1	ORF059L	1	1	8.70%	13.33	6.27
113	QLI60775.1	ORF011R	1	1	5.77%	12.07	7.98
